# Spatiotemporal Patterns of Five Sympatric Ungulates in the Tangjiahe National Nature Reserve, Sichuan, China

**DOI:** 10.3390/biology15191729

**Published:** 2026-10-01

**Authors:** Jiayuan Hu, Mei Xiao, Mingfu Li, Zhisong Yang, Yanfei Chen, Lingyu Liu, Jiao Liao, Weichao Zheng

**Affiliations:** 1 Sichuan Academy of Giant Panda, Chengdu 610081, China; hujiayuann@163.com (J.H.); yangzhisong@126.com (Z.Y.); yantel@163.com (Y.C.); 15228352379@163.com (L.L.); 18016123008@163.com (J.L.); 2 Sichuan Tangjiahe National Nature Reserve Administration Management, Guangyuan 628109, China; 15828439365@163.com (M.X.); lmfyy@163.com (M.L.)

**Keywords:** activity rhythm, camera trapping, niche differentiation, spatiotemporal niche, sympatric ungulates, Tangjiahe National Nature Reserve

## Abstract

Ungulates serve as a critical link between primary producers and higher-level consumers, and play an irreplaceable role in maintaining the structural and functional integrity of forest ecosystems. Understanding the spatiotemporal niche differentiation of sympatric ungulates is of great guiding significance for forest biodiversity conservation and refined habitat management. Based on camera-trapping data collected across the entire Tangjiahe National Nature Reserve from 2022 to 2024, this study systematically analyzed the relative detection intensity, multi-scale activity rhythms, spatiotemporal niche overlap characteristics, and environmental response patterns of five sympatric ungulate species: the Chinese takin (*Budorcas tibetana*), Chinese serow (*Capricornis milneedwardsii*), Chinese goral (*Naemorhedus griseus*), tufted deer (*Elaphodus cephalophus*), and forest musk deer (*Moschus berezovskii*). The results showed that the relative detection intensity of the five ungulates exhibited a three-tiered hierarchical pattern, with the Chinese takin showing the highest relative detection intensity and the Chinese serow and forest musk deer showing the lowest detection rates. Fitted diel activity curves were characterized mainly by crepuscular bimodal patterns, whereas forest musk deer showed a predominantly nocturnal unimodal pattern; the DI of Chinese serow showed no statistically supported overall bias between the predefined daytime and nighttime windows. Seasonal overlap coefficients and activity curves indicated species-specific seasonal adjustment, with Chinese serow showing the greatest cold–warm-season divergence and Chinese goral the highest seasonal similarity. The three widely distributed species displayed moderate to high spatiotemporal overlap, whereas the two narrowly distributed species showed pronounced differentiation in spatial-use patterns. Generalized additive models further revealed species-specific nonlinear associations with environmental variables, with elevation and distance to roads emerging as the most consistent predictors across all five species. These findings characterize the spatiotemporal niche differentiation pattern of sympatric ungulates in the Minshan Mountains, and offer a scientific basis for targeted monitoring and conservation of species with low detection rates, habitat zoning optimization, and seasonally adaptive regulation of human disturbance within the nature reserve.

## Simple Summary

Ungulates form a critical link between plants and predators and sustain the functioning of forest ecosystems. Clarifying spatiotemporal niche patterns among sympatric ungulates supports evidence-based biodiversity conservation. Based on 2022–2024 camera-trapping data from the Tangjiahe National Nature Reserve, this study investigated five co-occurring ungulate species: Chinese takin (*Budorcas tibetana*), Chinese serow (*Capricornis milneedwardsii*), Chinese goral (*Naemorhedus griseus*), tufted deer (*Elaphodus cephalophus*), and forest musk deer (*Moschus berezovskii*). The five species showed a stratified pattern of relative detection intensity: takin was the most frequently detected, while Chinese serow and forest musk deer were detected less frequently. Their fitted diel activity curves were characterized mainly by crepuscular bimodal patterns, whereas forest musk deer showed a predominantly nocturnal unimodal pattern. Seasonal activity patterns differed among species, with the greatest cold–warm-season divergence observed in Chinese serow and the highest seasonal similarity in Chinese goral. Three widespread species exhibited high spatiotemporal overlap, whereas two restricted-range species showed pronounced differentiation in spatial-use patterns. These observed spatiotemporal patterns align with the framework of multidimensional niche complementarity in ungulate communities. The findings provide scientific support for targeted ungulate conservation and refined habitat management in the reserve.

## 1. Introduction

The coexistence of sympatric species is a central question in community ecology and a key to understanding the maintenance of biodiversity [1,2,3]. The competitive exclusion principle predicts that species with completely identical niches cannot coexist stably over the long term [4], whereas Hutchinson’s multidimensional niche concept emphasizes that species may differ along temporal, spatial, trophic, and other resource axes [5]. Schoener (1974) subsequently identified habitat, food, and time as major dimensions of resource partitioning in animal communities [6]. Modern coexistence theory extends this framework by distinguishing stabilizing niche differences, which strengthen intraspecific relative to interspecific limitation, from relative fitness differences that favor some species over others; environmental filtering simultaneously constrains which species and ecological strategies can persist under local abiotic conditions [1,7]. Consequently, observed spatial or temporal separation may be consistent with niche partitioning, but it does not by itself demonstrate competition or a stabilizing coexistence mechanism.

Ungulates are among the most abundant and widely distributed mammalian groups in forest ecosystems. As a critical link between primary producers and higher-level consumers, they play an irreplaceable role in maintaining the structural and functional integrity of ecosystems [8,9]. On the one hand, as primary consumers, ungulates regulate the species composition, vertical structure, and regeneration dynamics of plant communities through foraging, trampling, and nutrient return via feces, making them an important functional group in forest ecosystems [10]. On the other hand, ungulates constitute the core prey base for large carnivores such as tigers, leopards, and dholes; their population abundance and distribution patterns directly determine the population persistence and spatial distribution of apex predators, thus serving as the foundation for maintaining the integrity of terrestrial food chains and the stability of food webs [11]. Meanwhile, most ungulate species are highly sensitive to habitat changes and anthropogenic disturbances, making them important indicator taxa for assessing forest ecosystem health [12,13]. Therefore, systematically analyzing the spatiotemporal niche differentiation and species coexistence patterns of sympatric ungulates not only helps reveal the assembly rules of forest animal communities but also provides a scientific basis for the conservation of species of concern and the refined management of their habitats.

The Minshan Mountains are part of the Southwest China Mountains biodiversity hotspot, one of the 25 global biodiversity hotspots, and harbor exceptionally high species richness and a large proportion of endemic species [14,15]. This region features complex topography, pronounced vertical climatic gradients, and a complete vegetation spectrum. It constitutes the core distribution area for numerous nationally protected wildlife species, including the giant panda (*Ailuropoda melanoleuca*), Chinese takin (*Budorcas tibetana*), and forest musk deer (*Moschus berezovskii*), and supports one of the most intact mammal community structures in China [16,17]. The Tangjiahe National Nature Reserve, located in the northern section of the Minshan Mountains, serves as a core habitat and gene flow corridor for wildlife populations in this mountain system. The reserve experiences low anthropogenic disturbance, retains well-preserved primary vegetation, and spans an elevational range exceeding 2700 m, encompassing a complete sequence of vertical habitats from evergreen broad-leaved forests to alpine shrub-meadows. These conditions support a rich assemblage of ungulate resources, including protected species such as the Chinese takin and forest musk deer. With high species richness, elevated protection status, and an intact community structure, the reserve provides an ideal setting for research on the coexistence of sympatric ungulates [16,18,19].

Previous studies in Tangjiahe have mainly addressed the population status, diet, habitat use, or activity of individual ungulate species [20,21,22,23,24,25]. A recent multi-species study compared activity rhythms and spatial use among sympatric artiodactyls [26], but an integrated, multi-year assessment linking standardized detection patterns with seasonal temporal overlap, spatial-use overlap, and nonlinear environmental responses has remained limited. This gap is important because overlap metrics alone cannot distinguish the effects of shared ecological requirements, environmental filtering, resource abundance, and interspecific interactions. Conversely, explicit analysis of elevation, slope, aspect, land cover, and distances to water and roads can provide an environmental context for interpreting observed distribution patterns, while still recognizing that camera-trap detections do not directly measure competition or demographic abundance.

Using camera-trap data collected throughout Tangjiahe from 2022 to 2024, we quantified relative detection intensity, diel and monthly activity rhythms, seasonal changes in activity, pairwise temporal and spatial-use overlap, and species-specific nonlinear associations with environmental predictors for five sympatric ungulates: Chinese takin, Chinese serow (*Capricornis milneedwardsii*), Chinese goral (*Naemorhedus griseus*), tufted deer (*Elaphodus cephalophus*), and forest musk deer. We evaluated three predictions. First, species detected broadly across the reserve were expected to show greater spatial-use and temporal overlap with one another than species with restricted detection distributions. Second, species with contrasting diel preferences were expected to show lower temporal overlap, whereas the magnitude of seasonal rhythm shifts was expected to vary among species. Third, environmental associations were expected to be species-specific, reflecting environmental filtering and differences in ecological requirements, although variables related to topography and anthropogenic access were expected to influence multiple species. By integrating these complementary analyses, this study aims to provide evidence relevant to hypotheses of niche partitioning and to identify species- and environment-specific priorities for ungulate conservation in Tangjiahe.

## 2. Materials and Methods

### 2.1. Study Area

The Tangjiahe National Nature Reserve (hereinafter referred to as the Tangjiahe Reserve) is located in Qingchuan County, Guangyuan City, Sichuan Province (104°36′–104°52′ E, 32°30′–32°41′ N), on the northern slopes of the Minshan Mountains. It extends approximately 31.0 km from east to west and 20.5 km from north to south (Figure 1) [27]. The reserve has a subtropical humid monsoon climate with pronounced vertical climatic zonation. The elevation ranges from 1150 to 3837 m, and the vertical vegetation spectrum is complete. From low to high elevations, vegetation types are sequentially evergreen broad-leaved forest, mixed evergreen–deciduous broad-leaved forest, mixed coniferous–broadleaved forest, subalpine coniferous forest, and alpine shrub-meadow, providing diverse habitats for wildlife. The reserve harbors abundant mammal resources and a structurally intact ungulate community, including several nationally protected species [28]. Based on the local climatic regime of Tangjiahe, November–March was classified as the cold season and April–October as the warm season. This division follows previous work in Tangjiahe, which defined the cold season as months characterized by lower mean temperature (≤10 °C), lower precipitation, and reduced sunshine, and the warm season as months with higher mean temperature (≥10 °C), greater precipitation, and stronger sunshine [26,27].

### 2.2. Camera Trap Deployment

From January 2022 to December 2024, a camera-trapping monitoring network was established using a systematic 1 km × 1 km fish-net grid framework [29]. In total, we achieved 792 camera-trap deployment events, corresponding to 592 unique independent camera stations. Some stations were retrieved for maintenance and redeployed during the three-year survey, which explains why total deployment events exceeded the number of unique stations. Monitoring stations were planned across the whole reserve following the grid system, but cameras could not be placed in grids inaccessible due to extremely steep terrain. For grids with high wildlife activity, 2–3 cameras were deployed within a single grid cell to improve detection probability, leading to uneven sampling density across grid cells. Cameras were mounted on tree trunks 30–80 cm above ground, facing animal trails and valley corridors while avoiding direct sunlight to reduce false triggering. Cameras operated in 24-h recording mode, capturing three consecutive photographs per trigger with a 1-s interval between triggers [30]. Habitat metadata including latitude, longitude, elevation, vegetation type, slope and aspect were recorded for each unique camera station during installation. Equipment maintenance and data download were performed every 3–6 months. Monitoring periods invalidated by equipment failure, damage or vegetation obstruction were excluded, yielding a cumulative total of 144,458 valid camera-days for subsequent analysis. Detailed sampling-effort statistics are provided in Supplementary Table S1. 

### 2.3. Data Sources and Processing

#### 2.3.1. Camera-Trap Data

All camera-trap images were reviewed, and species identification was conducted following the Atlas of Mammals of China, from which valid records of the five target ungulate species—Chinese takin, Chinese serow, Chinese goral, tufted deer, and forest musk deer—were extracted [31]. Following the widely adopted convention in camera-trapping studies, we operationally defined an independent detection event: records of the same species at a given camera station were merged into a single event if separated by less than 30 min [32,33]. This criterion removes short-interval burst-series duplicates from the dataset, but it does not guarantee strict statistical independence. The same individual or social group may be repeatedly recorded at the same site across longer time windows, introducing potential spatial autocorrelation among detection records. We used detection events as the base unit for kernel-density estimation of diel activity rhythms and overlap-coefficient calculation, while acknowledging that subsequent permutation-based inference tests carry inherent limitations due to potential non-independence of observations.

The effective monitoring days (i.e., the number of calendar days during which a camera was functioning properly) were calculated for each camera station, after excluding invalid monitoring days. These were then aggregated to obtain the total cumulative effective monitoring days across the entire reserve, as well as the number of independent detection events for each species in different years and seasons.

#### 2.3.2. Environmental Variables

Six environmental variables were used to characterize the topographic, land-cover, hydrological, and anthropogenic conditions associated with the spatial use of the five ungulate species: elevation, slope, aspect, land use/land cover (LUCC), distance to water, and distance to roads.

Topographic variables were derived from digital elevation model (DEM) data originating from the Shuttle Radar Topography Mission (SRTM) and distributed through WorldClim version 2.0. Elevation values were extracted for each camera station from the DEM, and slope and aspect were further calculated in ArcGIS 10.8. Elevation was expressed in meters above sea level, slope was expressed in degrees, and aspect was expressed as azimuth (0–360°), with north occurring at both 0° and 360°.

Hydrological conditions were represented by the minimum distance from each camera station to the nearest drainage feature. The drainage network was extracted from the DEM using standard hydrological procedures, including sink filling, flow-direction calculation, flow accumulation, and stream-network extraction, after which the nearest distance from each camera station to a drainage line was calculated in ArcGIS.

Land-use/land-cover data were sourced from the National Tibetan Plateau Data Center https://data.tpdc.ac.cn (accessed on 19 September 2026), with a resolution ranging from 10 to 100 m [34]. LUCC was retained as a categorical variable using the original land-cover codes represented within the study area. This variable therefore describes the land-cover context surrounding camera stations and was interpreted as an environmental association rather than direct evidence of habitat selection.

Anthropogenic disturbance was represented by distance to roads. Road-network vector layers covering the study area were merged into a single road-network dataset, and the minimum distance from each camera station to the nearest road was calculated using the Near analysis in ArcGIS. All environmental layers were projected to a common coordinate reference system before spatial extraction and distance calculation. The resulting environmental values were linked to the unique camera stations and subsequently used in species-specific GAM analyses.

### 2.4. Data Analysis

#### 2.4.1. Relative Abundance Index

The relative abundance index (RAI) was calculated as an effort-standardized index of relative detection intensity among the five ungulate species in the Tangjiahe Reserve [35,36]:

$$\;RAI=A_{i}∕T\times 100$$

where Aᵢ represents the number of independent detection events (photographs or videos) for species i (i = 1, 2, 3, …), and T represents the total number of camera-trap days. Independent detection events (photographs or videos) must satisfy the following three conditions: (1) consecutive valid records involve different individuals of the same or different species; (2) the interval between consecutive valid records containing individuals of the same species is at least 30 min; (3) non-consecutive valid records contain individuals of the same species. Annual RAI values were computed separately for each calendar year, using year-specific total effective camera-days as the denominator. The overall three-year RAI was calculated using cumulative detection events and cumulative total effective camera-days across the entire study period, and is not the arithmetic mean of annual RAI values but a weighted average weighted by annual sampling effort.

#### 2.4.2. Diel Activity Characteristic Indices

The 24-h day was divided into daytime (06:00–18:00) and nighttime (18:00–06:00 the following day). These fixed clock-time intervals were used as an operational definition to ensure consistent calculation across the three-year dataset. Because actual sunrise and sunset vary seasonally, DI should be interpreted as the balance of activity between these fixed time windows rather than as a strict biological classification of diurnality or nocturnality. The following indices were calculated to quantitatively characterize the temporal allocation pattern and degree of balance in species’ diel activity:

(1) Diurnality Index (DI)

This index quantifies the direction and strength of activity bias between the predefined daytime and nighttime windows [37,38]. It was derived by integrating kernel-density activity probability density over the daytime and nighttime windows:

$$DI=\frac{D_{area}-N_{area}}{D_{area}+N_{area}}$$

where D_area_ is the integrated activity probability density during daytime, and N_area_ is the integrated activity probability density during nighttime. DI ranges from −1 to 1, with positive values indicating greater activity within the predefined daytime window, negative values indicating greater activity within the predefined nighttime window, and larger absolute values denoting a stronger diel-activity bias between these two clock-time periods. To assess estimation uncertainty, especially for rare species with limited detections, 95% non-parametric percentile bootstrap confidence intervals were generated by resampling raw independent detection events with replacement (1000 replicates).

(2) Shannon–Wiener diversity index (H) and maximum Shannon index (H_max_)

To calculate the evenness of a species’ daily activity rhythm, the 24-h day was first divided into 24 equal time intervals (hours). The Shannon–Wiener diversity index (H) characterizes the degree of dispersion of a species’ activity across the 24 time intervals [39], and is given by:

$$H=-\sum_{i=1}^{S}P_{i}lnP_{i}$$

where S is the number of time intervals (i.e., 24), and Pᵢ is the proportion of independent detection events occurring in the i-th time interval relative to the total detection events for the day. The theoretical maximum Shannon index ($H_{max}$) is:

$$H_{max}=lnS$$

(3) Evenness index (J)

This index is used to measure the degree of uniformity in the allocation of species activity across the 24-h time axis [40], and is calculated as:

$$J=\frac{H}{H_{max}}$$

J ranges from 0 to 1. A J value closer to 1 indicates a more uniform distribution of daily activity without distinct peaks and troughs; a smaller J value indicates that activity is more concentrated within a specific time period.

(4) Daily activity concentration index (DDI)

In this study, DDI is defined as the complement of the evenness index of activity distribution across the full 24-h cycle, used to quantify the degree of temporal clustering of daily activity. It is derived directly from the evenness index J [40]:

DDI=1−J

This index ranges from 0 to 1. A higher DDI value indicates that daily activity is more concentrated in specific time periods; a DDI value approaching 0 indicates that activity is more evenly distributed across the 24-h cycle.

#### 2.4.3. Temporal Activity Rhythm Analysis

Kernel density estimation was used to fit the 24-h daily activity rhythm curves of the five ungulate species [41], identify activity peaks and troughs, and classify activity pattern types. In this study, R 4.6.0 software was used, and kernel density fitting of daily activity rhythms was performed using the fitact function from the activity package to obtain smoothed activity density curves. Independent event counts for each species were tabulated by month, and the same kernel density estimation method was applied to fit annual activity rhythm curves to analyze annual activity fluctuation patterns and peak displacement characteristics.

Kernel density curves of daily activity rhythms were fitted separately for the cold and warm seasons. The coefficient of overlap Δseason was used to quantify the similarity between cold- and warm-season rhythms for the same species [41]. Δseason ranges from 0 to 1, with higher values indicating greater rhythm overlap and smaller seasonal differences. The overlap coefficient was calculated using the overlapEst function from the overlap package [42], with the estimator dynamically selected based on sample size: the Δ_4_ estimator was used when the number of independent detection events in both groups was ≥75; the Δ_1_ estimator was used when the sample size of either group was <75. The compareCkern function from the activity package [43] was used to run 1000-iteration permutation tests for seasonal differences in diel-activity rhythms. This permutation approach assumes exchangeable, statistically independent observations, an assumption that may be violated by spatial autocorrelation among detection events noted above. We therefore treat the resulting *p*-values as suggestive rather than conventional inferential evidence and place primary emphasis on Δseason values and observed changes in the fitted activity curves; α = 0.05 is retained only as a nominal reference threshold.

#### 2.4.4. Temporal Niche Overlap Analysis

Based on the daily activity rhythm kernel density curves for the cold and warm seasons, the temporal niche overlap coefficient Δspecies between pairs of species was calculated using exactly the same estimation rules and testing methods as described above to evaluate the degree of temporal niche overlap between species in different seasons. Following commonly used classification criteria, overlap levels were defined as: low overlap (Δspecies < 0.5), moderate overlap (0.5 ≤ Δspecies < 0.8), and high overlap (Δspecies ≥ 0.8) [44].

#### 2.4.5. Spatial Distribution and Spatial Niche Overlap Analysis

We divided the entire reserve into 1 km × 1 km monitoring grids using a fishnet framework. To correct for uneven sampling effort across grid cells caused by variable camera deployment density and operational duration, we calculated the relative detection rate (RDR) for each species per grid as a standardized metric of space-use intensity [45]:

$$RDR=\frac{N_{grid}}{{EC}_{grid}}\times 100$$

where N_grid_ is the number of independent detection events of a given species within the grid, and EC_grid_ is the total cumulative effective camera-days across all camera stations deployed within that grid. For camera stations redeployed at the same geographic location, detections and valid camera-days from all deployment periods were accumulated before grid-level aggregation. Sampled grids with zero detections were assigned RDR = 0, whereas grids without camera deployment were treated as unsampled and excluded from RDR calculation and Schoener’s D estimation. We classified RDR values into five ordinal categories (no detection, low, medium, relatively high, and high) using the Jenks natural breaks classification method for spatial visualization and defined grids with RDR values falling in the upper quartile of all grids with detections as species distribution hotspots.

To quantify pairwise spatial-use overlap while accounting for differences in use intensity rather than just presence–absence, we calculated Schoener’s D niche overlap index based on grid-level RDR distributions [46,47]. First, RDR values for each species were normalized across all sampled grids to produce proportional space-use vectors:

$$p_{i}=\frac{{RDR}_{i}}{\sum RDR},\;q_{i}=\frac{{RDR\prime }_{i}}{\sum RDR\prime }$$

where RDR*_i_* and RDR*_i_*′ are the RDR values of the two focal species in grid i, and ∑RDR and ∑RDR′ are the sum of RDR across all monitored grids for each species. Schoener’s D was then computed as:

$$\;D=1-0.5\sum_{i=1}^{n}\left|pi\right.-\left.qi\right|$$

D ranges from 0 (no overlap, completely segregated space use) to 1 (complete overlap, identical space-use intensity distributions). We categorized overlap magnitude following the standardized five-tier classification scheme for Grinnellian niche overlap proposed by Rödder and Engler (2011): no or very limited overlap (0 ≤ D < 0.20), low overlap (0.20 ≤ D < 0.41), moderate overlap (0.41 ≤ D < 0.61), high overlap (0.61 ≤ D < 0.81), and very high overlap (0.81 ≤ D ≤ 1.0) [48].

#### 2.4.6. Generalized Additive Model Analysis

Generalized additive models (GAMs) were used to quantify potentially nonlinear relationships between environmental variables and species-specific detection intensity. GAMs were selected because they allow flexible estimation of nonlinear ecological responses without requiring a predetermined parametric form for each continuous predictor [49,50].

For each species, independent detection events were summarized at the camera-site-year level. The model-ready dataset contained 792 camera-site-year observations, and zero-detection observations were retained for all species. Species-specific numbers and proportions of zero-detection observations are provided in Supplementary Table S3. The response variable was the site-year detection-event count. The six environmental predictors were elevation, slope, aspect, LUCC, distance to water, and distance to roads. Elevation, slope, distance to water, and distance to roads were fitted as penalized smooth terms. Because aspect is a circular variable, it was fitted using a cyclic cubic regression spline to ensure continuity between 0° and 360°. LUCC was treated as a categorical factor. Year was included as a categorical covariate to account for interannual variation during the 2022–2024 monitoring period.

The general model structure was: detection intensity = β0 + s(elevation) + s(slope) + s(aspect) + LUCC + s(distance to water) + s(distance to roads) + year + ε, where β0 is the intercept and s(·) denotes a penalized smooth function. A negative binomial error distribution with a log link was used to accommodate overdispersion. Continuous smooth terms were constrained to low complexity (k = 5), and the aspect was fitted with a cyclic spline. Smoothing parameters were estimated automatically to balance model fit and complexity and thereby reduce overfitting. The contribution of each environmental predictor was evaluated by comparing the full model with a reduced model excluding the focal term using likelihood-ratio tests. Statistical significance was assessed at α = 0.05. Model explanatory performance was summarized as the percentage of deviance explained.

For visualization, fitted responses were predicted across the observed range of each continuous environmental variable while the remaining predictors were held constant. The fitted line represents the model-predicted detection intensity, and the shaded band represents the corresponding 95% confidence interval. For LUCC, predicted means and 95% confidence intervals were displayed for each categorical land-cover code. All GAM analyses and response-curve visualizations were conducted in R using the mgcv framework [50,51].

## 3. Results

### 3.1. Characteristics of Relative Detection Intensity

During the monitoring period from 2022 to 2024, camera traps across the reserve accumulated a total of 144,458 effective camera-days, yielding 40,794 independent detection events of the five target ungulate species. Calculation of the relative detection index (RAI) revealed clear differences in relative detection intensity among species. The overall three-year RAI ranked in descending order: Chinese takin > Chinese goral > tufted deer > Chinese serow > forest musk deer.

Chinese takin showed the highest relative detection intensity, with annual RAI values of 11.91, 8.02, and 18.81 detections per 100 camera-days in 2022, 2023, and 2024, respectively. Its overall RAI remained the highest among the five species throughout the study period. Chinese goral and tufted deer formed the second detection tier, with overall three-year RAI values of 8.89 and 4.07 detections per 100 camera-days, respectively. Both species showed similar interannual trends, peaking in 2024.

Chinese serow and forest musk deer exhibited the lowest relative detection intensity, with overall three-year RAI values below 0.2 detections per 100 camera-days. The RAI of Chinese serow remained stable across years, while forest musk deer showed a gradually increasing annual trend. (Table 1 and Table S2).

### 3.2. Diel Activity Characteristic Indices of the Five Ungulate Species

Based on the integrated monitoring data from 2022 to 2024, the diurnality index (DI) and daily activity difference index (DDI) were calculated for the five ungulate species. The results revealed distinct differentiation in diel activity habits among the species.

#### 3.2.1. Diurnality Index

The diurnality index (DI) together with 95% bootstrap confidence intervals revealed clear divergence in diel-activity patterns among the five sympatric ungulate species.

Three species showed statistically supported positive DI values, indicating greater activity within the predefined daytime window: Chinese takin (DI = 0.320, 95% CI: 0.308–0.332), Chinese goral (DI = 0.190, 95% CI: 0.174–0.206), and tufted deer (DI = 0.283, 95% CI: 0.260–0.306). Forest musk deer showed a statistically supported negative DI value (DI = −0.253, 95% CI: −0.423–−0.084), indicating greater activity within the predefined nighttime window. Chinese serow showed a negative DI estimate (DI = −0.106), but its 95% CI spanned zero (−0.236–0.027), indicating no statistically supported overall bias between the predefined daytime and nighttime windows (Table 2).

#### 3.2.2. Daily Activity Difference Index

The daily activity difference index (DDI) results showed variation in the temporal concentration of activity among the five ungulate species. The forest musk deer had the highest DDI (0.130), indicating that its activity was the most concentrated within a relatively narrow portion of the 24-h cycle. This was followed by the Chinese takin (DDI = 0.038), tufted deer (DDI = 0.031), and Chinese serow (DDI = 0.029), all of which showed relatively low temporal concentration. The Chinese goral had the lowest DDI (0.012), indicating the most even distribution of activity across the 24-h cycle (Table 2).

### 3.3. Multi-Scale Temporal Activity Rhythms of the Five Ungulate Species

#### 3.3.1. Daily Activity Rhythms of Individual Species

The fitted diel activity curves of the five ungulate species showed pronounced interspecific differences and could be broadly characterized as crepuscular bimodal or predominantly nocturnal unimodal patterns. Among them, the Chinese takin, Chinese goral, tufted deer, and Chinese serow all exhibited crepuscular bimodal activity rhythms, with daily activity curves displaying two distinct activity peaks in the early morning (06:00–08:00) and late afternoon (18:00–20:00), while the periods of 10:00–16:00 during the day and 00:00–04:00 at night represented activity troughs. The forest musk deer showed a predominantly nocturnal unimodal activity rhythm, with its daily activity curve exhibiting only one distinct activity peak, concentrated during the nighttime period of 18:00–20:00, and very low activity during the predefined daytime window (Figure 2).

#### 3.3.2. Monthly Activity Rhythms

The annual activity rhythms of the five ungulate species exhibited pronounced seasonal fluctuations, with differentiation in the active periods among species. The Chinese takin showed a bimodal annual activity pattern, with peaks occurring in April–May and October–November and troughs in January–February and July–August. The Chinese goral also exhibited a bimodal annual pattern, with activity peaks concentrated in May and October and troughs in March and December. The tufted deer displayed a unimodal annual activity pattern, peaking in May–June and with troughs in January–March and November–December. The Chinese serow’s annual activity peaked in September, with low activity intensity throughout the remaining months and no obvious secondary peak. The forest musk deer showed a bimodal annual activity pattern, with peaks in May and October, highly overlapping with the activity rhythm of the Chinese goral, and troughs in January–February and December (Figure 3).

#### 3.3.3. Seasonal Differences in Daily Activity Rhythms

After dividing the year into cold and warm seasons based on the climatic characteristics of the reserve, we compared the daily activity rhythms of the five ungulate species between the two seasons. The results showed that, with the exception of the forest musk deer, permutation analyses indicated potential seasonal shifts in diel activity rhythms for the other four species (raw permutation *p* < 0.001; *p*-values may be biased due to spatial autocorrelation among detections; Figure 4).

For the Chinese takin, Chinese goral, tufted deer, and Chinese serow, the fitted activity curves showed seasonal changes in peak timing and shape, with warm-season peaks generally occurring earlier than in the cold season. Among them, Chinese goral had the highest seasonal rhythm overlap coefficient Δseason = 0.939, indicating high similarity between cold- and warm-season activity rhythms, followed by the Chinese takin (Δseason = 0.866), tufted deer (Δseason = 0.827), and Chinese serow (Δseason = 0.667). The lower Δseason value of Chinese serow reflected relatively larger seasonal rhythm divergence. The forest musk deer retained a broadly similar seasonal activity pattern (Δseason = 0.814); its nocturnal activity pattern remained relatively stable across both seasons, showing no marked seasonal adaptive adjustments (Figure 4). Permutation *p*-values are reported only as suggestive results because repeated detections may violate the exchangeability assumption.

### 3.4. Analysis of Temporal Niche Overlap Between Species During the Cold and Warm Seasons

Based on the monitoring data from 2022 to 2024, pairwise temporal niche overlap indices were calculated for the five ungulate species in both the cold and warm seasons. The results showed that overall temporal overlap among species was higher in the warm season than in the cold season, with notable differences in the degree of overlap among different species pairs.

#### 3.4.1. Temporal Niche Overlap in the Cold Season

In the cold season, the pairwise temporal niche overlap indices among the five ungulate species ranged from 0.622 to 0.894, indicating a moderate-to-high overall overlap level. The highest overlap was observed between the Chinese takin and tufted deer (Δspecies = 0.894), followed by the Chinese takin and Chinese goral (Δspecies = 0.882) and the Chinese goral and tufted deer (Δspecies = 0.866). All three species pairs exhibited high overlap, with their activity rhythms being highly coincident during the cold season. The lowest overlap was found between the Chinese serow and tufted deer (Δspecies = 0.622), while the remaining species pairs fell within the range of 0.650 to 0.750, representing a moderate overlap level (Figure 5A).

#### 3.4.2. Temporal Niche Overlap in the Warm Season

In the warm season, the pairwise temporal niche overlap indices ranged from 0.607 to 0.927, showing a markedly higher overall overlap level than in the cold season, with a greater number of species pairs exhibiting high overlap. The Chinese takin and tufted deer again had the highest overlap (Δspecies = 0.927), followed by the Chinese goral and tufted deer (Δspecies = 0.907) and the Chinese takin and Chinese goral (Δspecies = 0.901). The overlap values for these three species pairs all exceeded 0.90, reaching an extremely high level, and their activity rhythms were almost completely coincident during the warm season. The lowest overlap occurred between the Chinese serow and forest musk deer (Δspecies = 0.607). The remaining species pairs showed overlap values ranging from 0.650 to 0.850, indicating moderate-to-high overlap levels (Figure 5B).

Overall, temporal overlap among the three species with statistically supported positive DI values—Chinese takin, Chinese goral, and tufted deer—remained consistently high in both seasons. Forest musk deer, which showed a statistically supported negative DI value and a predominantly nocturnal activity curve, generally exhibited lower temporal overlap with these three species. Chinese serow occupied an intermediate temporal position: its activity curve was crepuscular and bimodal, whereas its DI showed no statistically supported overall bias between the predefined daytime and nighttime windows.

### 3.5. Spatial Distribution Patterns Based on Standardized Relative Detection Rate

Based on the fishnet grid framework and sampling-effort standardization, we mapped the relative detection rate (RDR) of each ungulate species across the Tangjiahe Reserve (Figure 6). The five species exhibited pronounced differences in spatial distribution range and use intensity and could be broadly categorized into two distribution types: widespread with aggregated hotspots and narrow-scattered distribution.

#### 3.5.1. Widespread-Aggregated Species

The Chinese takin, Chinese goral, and tufted deer exhibited typical widespread-aggregated spatial patterns. Their distributions covered the majority of monitored grids across the reserve, with multiple distinct RDR hotspots. Chinese takin had the broadest distribution range, with core hotspot areas concentrated in the central and northern parts of the reserve. The core hotspot areas of Chinese goral and tufted deer were largely congruent with those of Chinese takin, mainly clustered in the central reserve, while low-to-moderate RDR values were also widely but more scattered across southern and eastern sections. All three species showed clear aggregated characteristics in their space-use intensity (Figure 6A,C,D).

#### 3.5.2. Narrow-Scattered Species

The Chinese serow and forest musk deer exhibited typical narrow, scattered spatial distributions. Their detection was restricted to a small subset of monitored grids, with no large continuous hotspot zones, forming an overall fragmented, patchy distribution pattern. Specifically, Chinese serow was concentrated in only a few grids in the central part of the reserve, with generally low RDR values. Forest musk deer occurred in isolated grids across the northern, central, and southern parts of the reserve, with no continuous distribution range and the lowest overall RDR among the five species (Figure 6B,E).

### 3.6. Spatial Niche Overlap Based on Schoener’s D Index

Pairwise Schoener’s D spatial-use overlap indices among the five ungulate species ranged from 0.065 to 0.635 (Figure 7). No species pair reached the threshold for very high overlap (D ≥ 0.81). The highest spatial-use overlap was observed between Chinese goral and Chinese takin (D = 0.635), which fell into the high overlap category (0.61 ≤ D < 0.81). Two species pairs fell into the moderate overlap range (0.41 ≤ D < 0.61): Chinese goral and tufted deer (D = 0.505) and tufted deer and Chinese takin (D = 0.502). Four species pairs were classified as low overlap (0.20 ≤ D < 0.41): forest musk deer and tufted deer (D = 0.212), forest musk deer and Chinese goral (D = 0.221), forest musk deer and Chinese takin (D = 0.231), and Chinese goral and Chinese serow (D = 0.254). The remaining three pairs showed no or very limited overlap (D < 0.20), with the lowest value occurring between forest musk deer and Chinese serow (D = 0.065), indicating nearly complete spatial segregation between the two less frequently detected species (Figure 7).

Overall, the three widespread-aggregated species (Chinese takin, Chinese goral, and tufted deer) showed moderate to high spatial-use overlap with one another, with largely coincident core habitat ranges. In contrast, the two species with more restricted detection distributions (Chinese serow, forest musk deer) exhibited no or very limited spatial overlap not only with the widespread species but also with each other, indicating pronounced differentiation in spatial-use patterns.

### 3.7. Environmental Responses Based on Generalized Additive Models

Generalized additive models revealed clear interspecific differences in environmental associations among the five ungulate species (Table 3). The proportion of deviance explained by the species-specific models ranged from 18.5% to 53.4%, with the highest value observed for Chinese serow (53.4%), followed by forest musk deer (32.1%), Chinese goral (20.2%), tufted deer (18.6%), and Chinese takin (18.5%).

Elevation and distance to roads were significantly associated with detection intensity for all five species. Elevation effects were significant for Chinese takin (*p* = 0.016), Chinese serow (*p* < 0.001), Chinese goral (*p* < 0.001), tufted deer (*p* < 0.001), and forest musk deer (*p* = 0.040). Similarly, distance to roads was significant for Chinese takin (*p* < 0.001), Chinese serow (*p* < 0.001), Chinese goral (*p* < 0.001), tufted deer (*p* < 0.001), and forest musk deer (*p* = 0.002).

Aspect was significantly associated with detection intensity in Chinese takin (*p* = 0.005), Chinese goral (*p* < 0.001), tufted deer (*p* < 0.001), and forest musk deer (*p* = 0.010), but not in Chinese serow (*p* = 0.286). Slope showed a significant effect only in Chinese serow (*p* = 0.021), whereas no significant effects were detected in Chinese takin (*p* = 0.133), Chinese goral (*p* = 0.268), tufted deer (*p* = 0.138), or forest musk deer (*p* = 0.159).

LUCC was significantly associated with detection intensity in Chinese takin (*p* < 0.001), Chinese serow (*p* < 0.001), and Chinese goral (*p* = 0.003), but not in tufted deer (*p* = 0.097) or forest musk deer (*p* = 0.249). Distance to water was significant for Chinese serow (*p* = 0.013) and forest musk deer (*p* = 0.011), whereas no significant effects were detected for Chinese takin (*p* = 0.209), Chinese goral (*p* = 0.096), or tufted deer (*p* = 0.245).

Significant interannual variation was detected for Chinese takin (*p* < 0.001), Chinese goral (*p* < 0.001), tufted deer (*p* < 0.001), and forest musk deer (*p* = 0.004), whereas the year effect was not significant for Chinese serow (*p* = 0.176). Overall, the GAM results indicate that elevation and road distance were the most consistently associated environmental predictors across the five species, while the effects of slope, aspect, LUCC, distance to water, and survey year varied among species. Detailed species-specific GAM response curves are provided in the Supplementary Materials (Figures S1–S6).

## 4. Discussion

### 4.1. Hierarchical Pattern of Relative Detection Intensity and Potential Drivers

The five ungulates showed a clear hierarchy in relative detection intensity. Chinese takin had the highest RAI, Chinese goral and tufted deer formed an intermediate group, and Chinese serow and forest musk deer were detected least frequently. This pattern was broadly similar to that reported from Wanglang Reserve in the Minshan Mountains [44], suggesting that comparable detection hierarchies may occur in parts of the northeastern Minshan region. Because camera-trap detection rates are also influenced by body size, movement rate, habitat use, trail use and behavioral wariness, however, the observed ranking should be interpreted as variation in relative detection intensity rather than direct differences in population density [52,53].

Chinese takin was widely recorded across the monitored landscape, which is consistent with its broad diet and use of a range of mountain forest habitats reported in previous studies [54,55]. Long-term protection may also have helped maintain suitable habitat conditions and connectivity within the reserve. Chinese goral and tufted deer likewise occurred across relatively broad areas. Previous habitat studies have shown that both species can use several forest types, although their vegetation associations are not identical [56]. Their comparatively frequent detections may therefore be related to broad habitat use and flexible movement across heterogeneous forest environments.

Chinese serow and forest musk deer were recorded less frequently and in more restricted parts of the camera network. The serow result is consistent with previous reports of its association with steep and concealed forest terrain [57], while the GAM also identified slope as an important environmental correlate for this species. Forest musk deer likewise showed low relative detection intensity and a scattered spatial distribution. These patterns justify targeted monitoring of both species, particularly in environmental conditions associated with their detections, but do not by themselves establish low population size.

Relative detection intensity also varied among years, most visibly in Chinese takin. Year remained significant in the GAMs for Chinese takin, Chinese goral, tufted deer and forest musk deer, whereas the effect was not significant for Chinese serow. Earlier work in Tangjiahe has discussed carrying capacity and food limitation in takin [58], so density dependence is one possible explanation for interannual variation. Changes in vegetation conditions, local habitat use, movement routes and camera deployment may also contribute. The three-year series therefore provides evidence of temporal variability in detections, but longer standardized monitoring will be needed to determine whether these changes represent persistent population trends.

### 4.2. Temporal Niche Differentiation and Its Coexistence Implications

Bootstrap confidence intervals supported positive DI values for Chinese takin, Chinese goral and tufted deer, indicating greater activity within the predefined daytime window, whereas forest musk deer showed a supported negative DI value, indicating greater activity within the predefined nighttime window. Chinese serow had a negative DI estimate, but its 95% confidence interval included zero, indicating no statistically supported overall bias between the predefined daytime and nighttime windows. Its fitted activity curve nevertheless showed clear peaks around dawn and dusk, so it is better described as crepuscular with an unresolved overall diel bias than as strictly nocturnal. Because DI was calculated using fixed clock-time windows (06:00–18:00 versus 18:00–06:00), it should be interpreted together with the fitted activity curves rather than used alone to assign species to strict diurnal or nocturnal categories. Seasonal changes in sunrise and sunset can shift dawn and dusk relative to these fixed boundaries, particularly for crepuscular species. Consequently, activity around twilight may contribute to either the daytime or nighttime component of DI depending on season. In the present study, the positive DI values and confidence intervals entirely above zero for Chinese takin, Chinese goral, and tufted deer indicate greater activity within the predefined daytime window, whereas the negative DI and confidence interval entirely below zero for forest musk deer indicate greater activity within the predefined nighttime window. Chinese serow is more appropriately described as crepuscular because its activity curve showed distinct dawn and dusk peaks while its DI confidence interval overlapped zero.

Chinese takin, Chinese goral, tufted deer and Chinese serow all exhibited crepuscular bimodal curves, with major peaks in the early morning and late afternoon. Similar activity schedules have been reported for ungulates in other temperate mountain systems [59,60]. These periods may provide favorable combinations of temperature and foraging opportunity, while reduced midday movement may reflect rest, rumination or avoidance of thermal stress. The shared timing of these peaks also helps explain why Chinese takin, Chinese goral and tufted deer showed relatively high temporal overlap in both seasons.

Forest musk deer differed by showing a predominantly nocturnal unimodal activity pattern and the highest temporal concentration of activity, whereas Chinese goral showed the most even distribution across the 24-h cycle. This contrast is consistent with temporal differentiation reported among sympatric ungulates in the Qinling and Gongga Mountains [59,61]. Such diel separation may reduce temporal coincidence among species with contrasting activity schedules, although it does not by itself demonstrate competitive avoidance.

Monthly detections of Chinese takin, Chinese goral and forest musk deer peaked mainly in spring and autumn, whereas those of tufted deer peaked in late spring to early summer and those of Chinese serow in September. These differences may be associated with species-specific use of seasonal food resources and reproductive demands [60,62,63]. Seasonal movements across elevations and changes in trail use may also affect detection frequency, making it difficult to attribute these peaks specifically to increased feeding or reproductive activity.

### 4.3. Seasonal Plasticity in Activity Rhythms and Environmental Drivers

Chinese serow showed the greatest divergence in activity, whereas Chinese goral retained the highest similarity between seasons. Forest musk deer showed comparatively little seasonal change. These contrasts indicate substantial interspecific variation in temporal plasticity. Seasonal interpretation therefore relies primarily on the overlap coefficients and fitted curve shapes, whereas the permutation *p*-values are treated only as suggestive evidence.

For Chinese takin, Chinese goral, tufted deer and Chinese serow, the main activity peaks generally occurred earlier in the warm season than in the cold season. Wang et al. (2024) [64] reported comparable seasonal changes in sympatric ungulates, and studies from temperate forests on the eastern Qinghai–Tibet Plateau and in northern Japan likewise documented shifts in diel activity across seasons [65,66]. Longer daylight, warmer temperatures and changes in vegetation phenology may alter suitable movement and foraging periods, while energetic and reproductive demands may further modify the timing of activity [60,67,68]. The larger seasonal divergence in Chinese serow and the high similarity in Chinese goral suggest that species exposed to the same seasonal environment differ in temporal plasticity rather than responding uniformly.

Forest musk deer retained a relatively stable nocturnal activity schedule between seasons. Forest canopies can buffer temperature extremes and reduce variation in understory microclimates [69,70], which may favor temporal stability in species using sheltered forest environments. Studies of other mountain ungulates, including Alpine ibex, also show that thermal conditions can alter the balance between daytime and nighttime activity [71]. The Tangjiahe results are therefore consistent with species-specific responses to seasonal environmental change rather than a single general pattern shared by all mountain ungulates.

### 4.4. Spatiotemporal Niche Coupling and Environmental Differentiation

Chinese takin, Chinese goral and tufted deer showed moderate-to-high spatial-use overlap with one another, whereas overlap involving Chinese serow and forest musk deer was generally much lower. Thus, similar daily activity schedules did not translate into equivalent spatial-use patterns across the monitored landscape.

The GAM results indicate that environmental associations differed markedly among species. Elevation and distance to roads emerged as the most consistent gradients across the assemblage, whereas topographic, land-cover and water-related effects varied among species. This pattern suggests that the observed spatial differentiation is associated with multiple components of environmental heterogeneity rather than a single habitat axis.

Elevation was the most consistent topographic gradient. In a mountain reserve such as Tangjiahe, elevation covaries with temperature, vegetation structure and resource distribution, while slope and aspect further modify accessibility, solar exposure and local vegetation. The significant elevation responses observed across all five species are consistent with recent evidence that elevational gradients strongly structure mountain ungulate distributions in southwestern China [72]. Chinese serow showed particularly strong environmental structuring, with its detections associated with several topographic, land-cover, hydrological and road-related variables, whereas forest musk deer showed a different combination of environmental associations. Their contrasting environmental responses are consistent with the low spatial overlap observed between these two species.

Distance to roads was also significant for every species. Roads may represent human access and disturbance, but they can also coincide with valley bottoms, altered vegetation and animal travel routes. The species-specific nonlinear GAM responses therefore suggest that road effects are more complex than simple avoidance. Even so, the consistency of the road-distance association across all five species identifies road-related gradients as an important component of the ungulate landscape and a priority for future monitoring.

Temporal overlap among Chinese takin, Chinese goral and tufted deer remained high in both seasons and generally increased in the warm season. Previous studies have proposed that reduced resource availability in colder periods can promote stronger temporal differentiation among sympatric ungulates [64], and the direction observed here is consistent with that hypothesis. Where temporal and spatial overlap remains substantial, dietary differentiation could provide an additional axis of separation. Studies of ruminants and African herbivore assemblages have documented fine-scale differences in food use even when broad spatiotemporal overlap is high [73,74]. Similar dietary differentiation may occur among Chinese takin, Chinese goral and tufted deer in Tangjiahe, but this remains a hypothesis because diet was not measured in the present study.

Overall, the observed community pattern is consistent with multidimensional niche differentiation. Chinese takin, Chinese goral and tufted deer shared substantial portions of time and space, whereas Chinese serow and forest musk deer showed stronger spatial differentiation and distinct environmental associations. These complementary temporal, spatial and environmental patterns may contribute to sympatry, but the present results do not identify a single mechanism responsible for coexistence.

### 4.5. Conservation and Management Implications

The broad and partly overlapping detection areas of Chinese takin, Chinese goral and tufted deer indicate that maintaining connectivity among frequently used parts of the central and northern reserve could benefit several species simultaneously. Because their spatial-use distributions are not identical, management of these shared areas should also retain vegetation and microhabitat heterogeneity rather than focus on a single habitat condition.

Chinese serow and forest musk deer warrant more targeted monitoring because their detections were sparse and environmentally differentiated. For Chinese serow, surveys can prioritize sites spanning the combinations of elevation, slope, LUCC, water distance and road distance associated with detections. For forest musk deer, priority surveys can focus on elevation, aspect, water-distance and road-distance conditions corresponding to observed records. Repeated sampling of such environments would improve estimates of the extent and persistence of their distributions.

The consistent association with road distance across all five species makes road-related gradients particularly relevant to management. New access routes, tourism facilities, maintenance corridors and patrol design should consider where the species-specific GAM responses change most strongly along this gradient. This provides a more defensible basis for local planning than applying a single uniform road buffer to all ungulates.

Activity patterns can also inform the timing of field operations. Several species were recorded most frequently around dawn and dusk, while monthly peaks differed among species. In heavily used locations, maintenance or tourism activities could therefore be scheduled to avoid periods of highest detections for focal species. Continued monitoring should retain comparable camera locations and effort records among years so that future changes in RAI, spatial use and environmental associations can be evaluated against the present three-year baseline.

### 4.6. Limitations of This Study

RAI and RDR are based on camera detections and do not explicitly estimate species-specific detection probability. Differences in movement, body size, trail use and camera encounter rates therefore remain part of these indices even after standardization by camera-days. The present study consequently interprets them as relative detection metrics rather than direct estimates of population abundance.

Repeated detections from the same camera station are not fully independent. The 30-min rule reduces immediate duplicates but does not remove spatial autocorrelation or repeated site use. In addition, the permutation procedure used for seasonal activity comparisons assumes exchangeable observations; its *p*-values are therefore interpreted only as suggestive evidence. The current GAMs also do not include camera station as a random effect, so repeated site-year observations may underestimate uncertainty. Site-level resampling, GAMMs with the camera station as a random effect, or occupancy models would provide stronger treatment of repeated observations and imperfect detection in future analyses.

The fixed 06:00–18:00 definition of daytime does not account for seasonal variation in sunrise and sunset. This can affect the numerical DI value of species active near dawn and dusk, particularly Chinese serow, whose kernel curve is crepuscular while its DI confidence interval crosses zero. Accordingly, DI is interpreted as an overall clock-time tendency rather than a strict behavioral category and is considered jointly with the kernel-density curve and bootstrap confidence interval. Solar-time analyses would provide a useful sensitivity test for DI interpretation and seasonal activity comparisons. Grids in extremely steep and inaccessible terrain were also not sampled, which may underestimate the distribution of species associated with such habitats.

Finally, the study did not directly measure diet, forage availability, microclimate or interspecific interactions. These data would be required to distinguish more clearly among environmental filtering, resource partitioning and competition. Combining repeated camera surveys with vegetation, microclimate, and dietary and demographic data would allow the candidate explanations raised by the present results to be tested directly.

## 5. Conclusions

Based on three years of camera-trap monitoring, the five sympatric ungulates in Tangjiahe showed clear differences in relative detection intensity, diel and seasonal activity, spatial-use overlap, and environmental associations. Chinese takin had the highest relative detection intensity, followed by Chinese goral and tufted deer, whereas Chinese serow and forest musk deer were detected much less frequently. Chinese takin, Chinese goral, and tufted deer showed greater activity within the predefined daytime window and high temporal overlap, whereas forest musk deer showed greater activity within the predefined nighttime window together with a predominantly nocturnal activity curve. Chinese serow showed a crepuscular pattern with no statistically supported overall bias between the predefined daytime and nighttime windows. Seasonal adjustment also differed among species, with Chinese serow showing the greatest divergence between cold and warm seasons and forest musk deer retaining the most stable seasonal activity pattern. Spatial-use overlap among Chinese takin, Chinese goral, and tufted deer was moderate to high, whereas overlap involving Chinese serow and forest musk deer was generally lower. The GAM results further demonstrated species-specific environmental associations, with elevation and distance to roads emerging as the most consistent predictors across all five species.

Taken together, these findings indicate that sympatric ungulates in Tangjiahe differ along temporal, spatial, and environmental dimensions, providing evidence consistent with multidimensional niche differentiation while avoiding attribution to a single coexistence mechanism. From a conservation perspective, maintaining connectivity and environmental heterogeneity in areas used by multiple species should be combined with targeted surveys of Chinese serow and forest musk deer in the environmental settings associated with their detections. The consistent association with road distance across all species also highlights the need to consider species-specific responses to road-related gradients when planning access, tourism, maintenance, and long-term monitoring within the reserve.

## Figures and Tables

**Figure 1:**
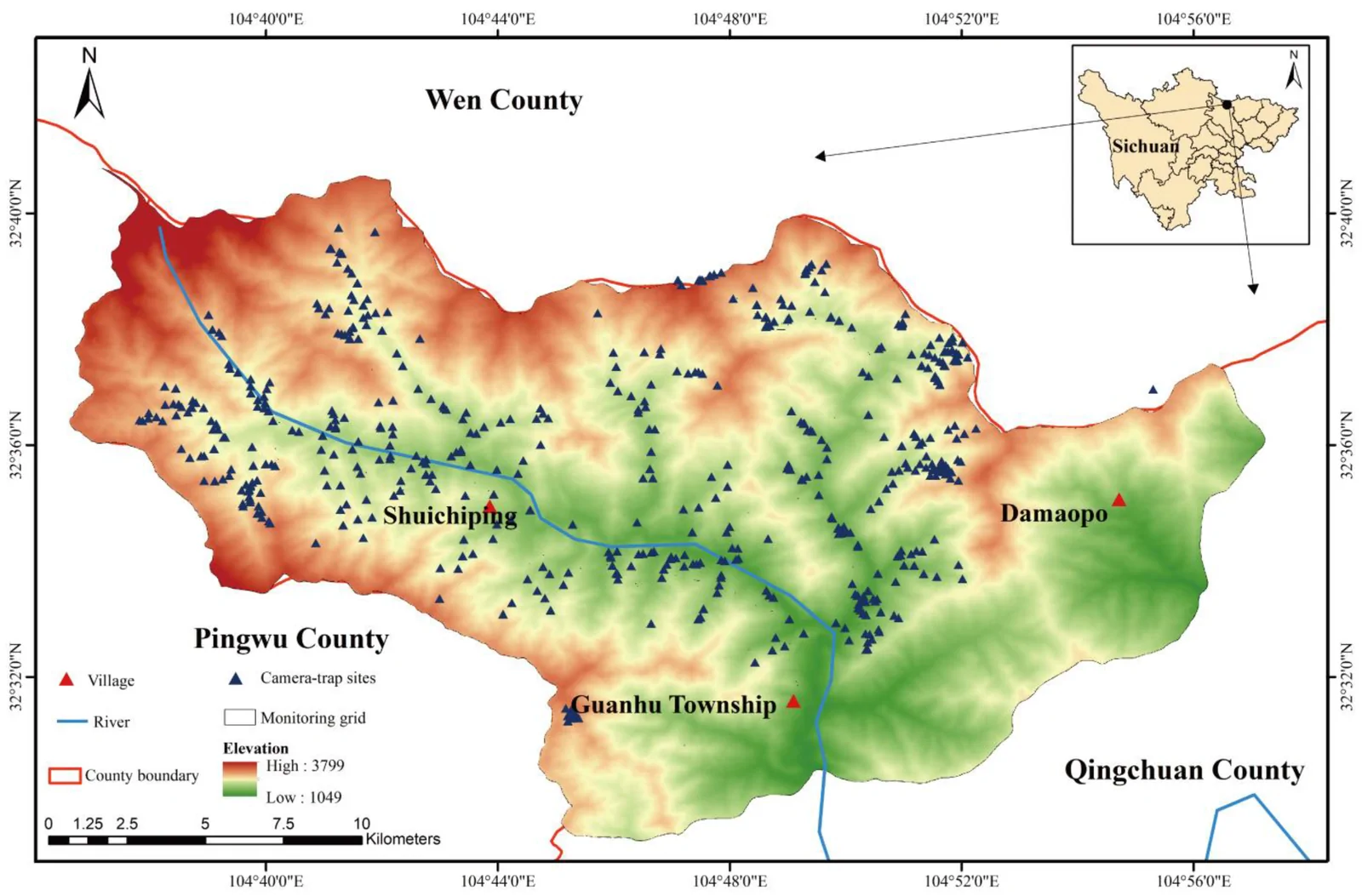
Overview Map of the Study Area.

**Figure 2:**
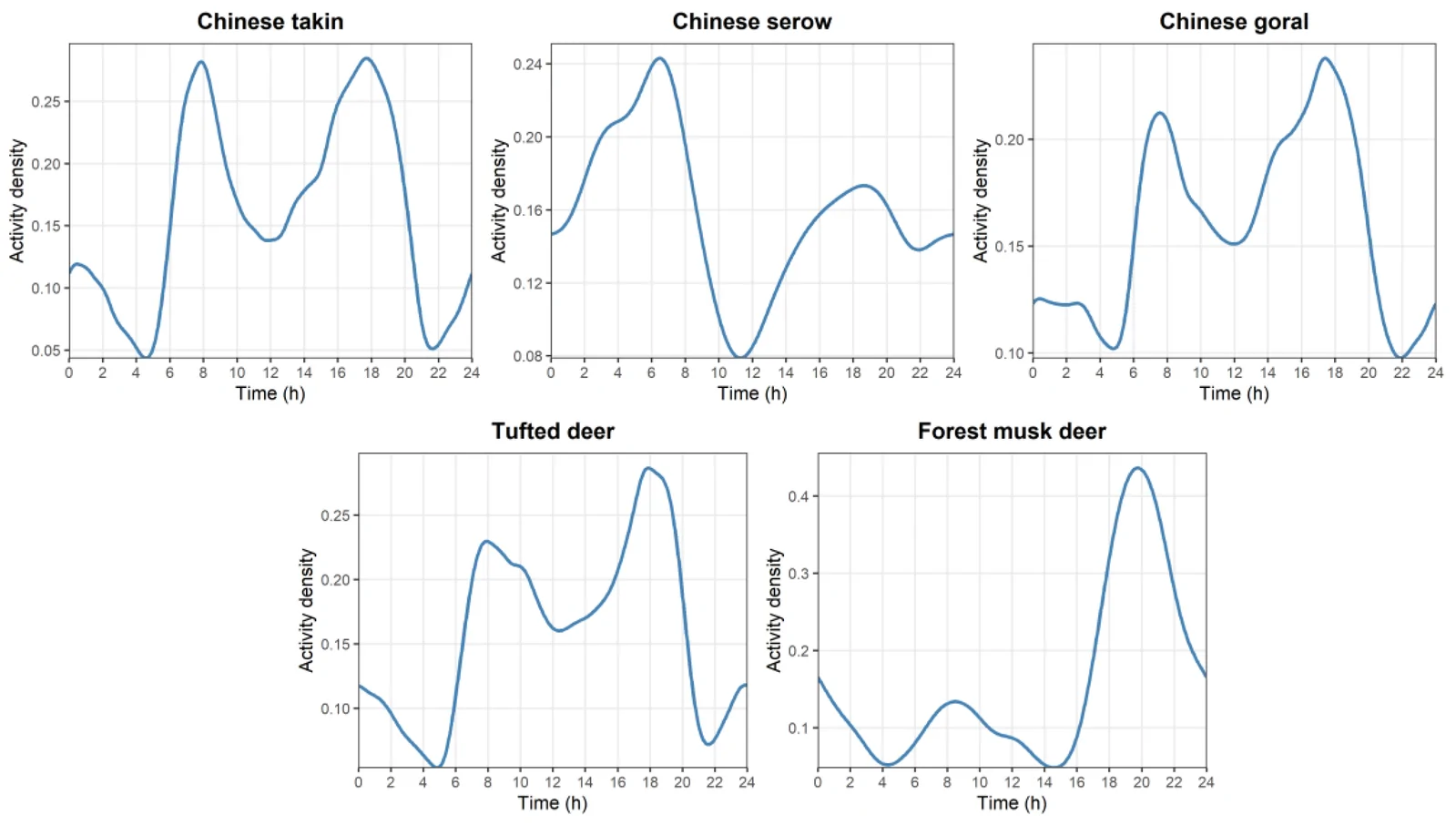
Daily activity rhythms of the five ungulate species in the Tangjiahe National Nature Reserve.

**Figure 3:**
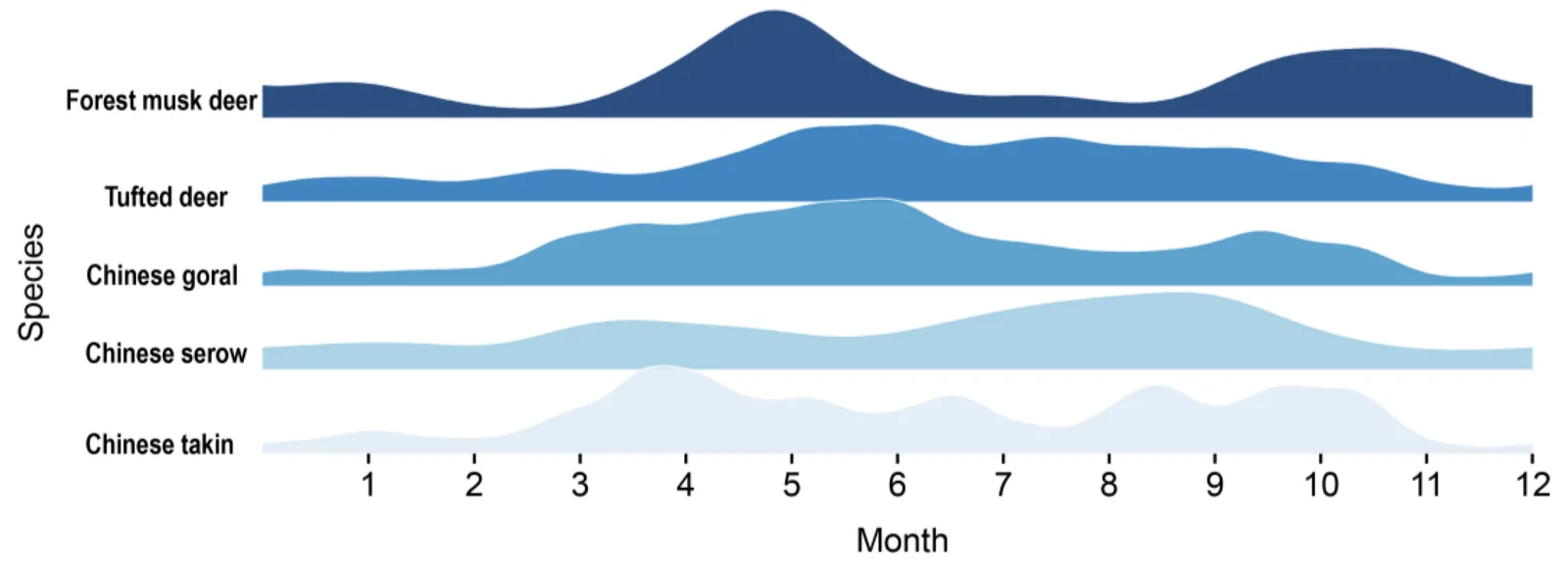
Monthly activity rhythms of the five ungulate species in the Tangjiahe National Nature Reserve.

**Figure 4:**
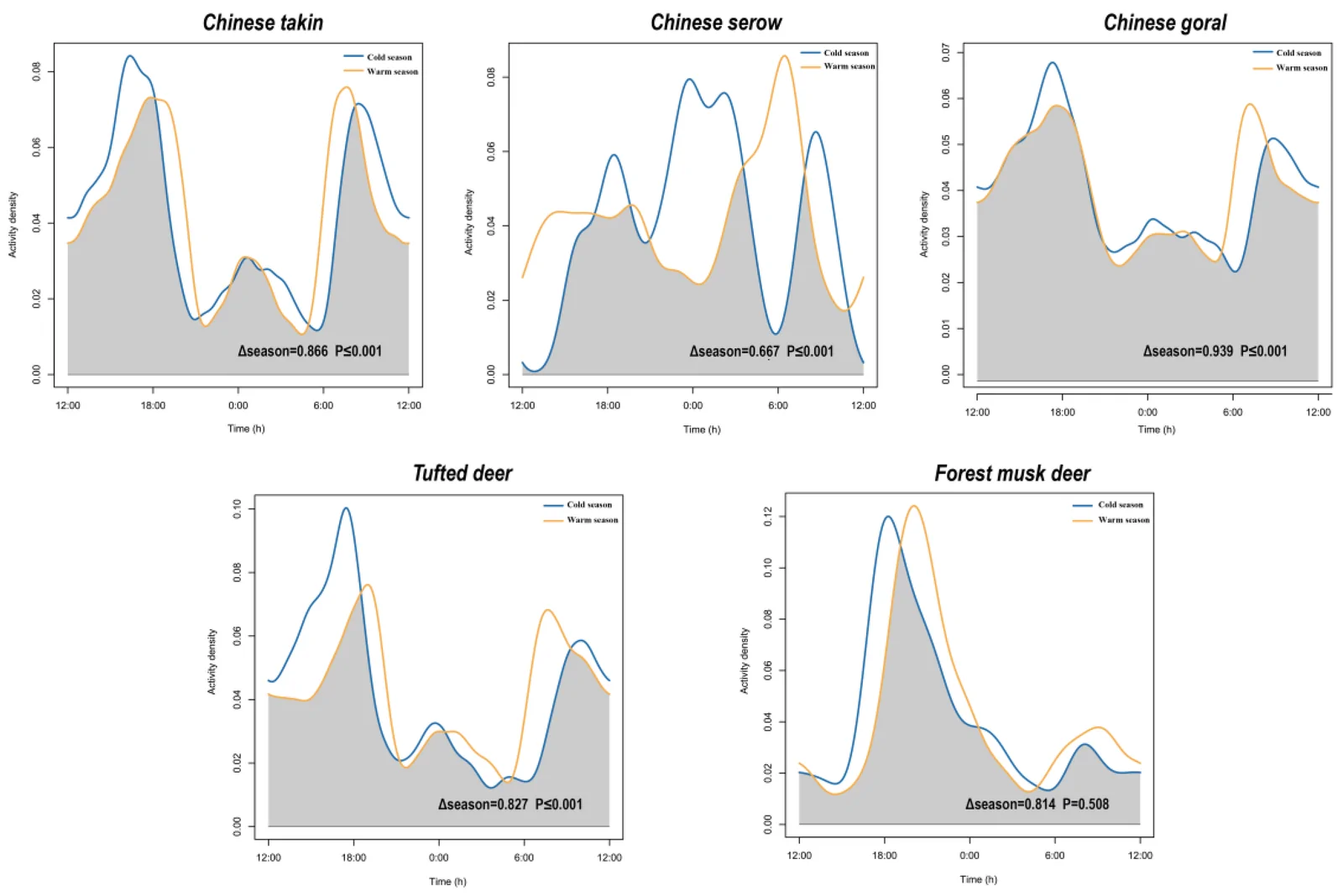
Daily activity rhythms of the five ungulate species during the cold and warm seasons in the Tangjiahe National Nature Reserve.

**Figure 5:**
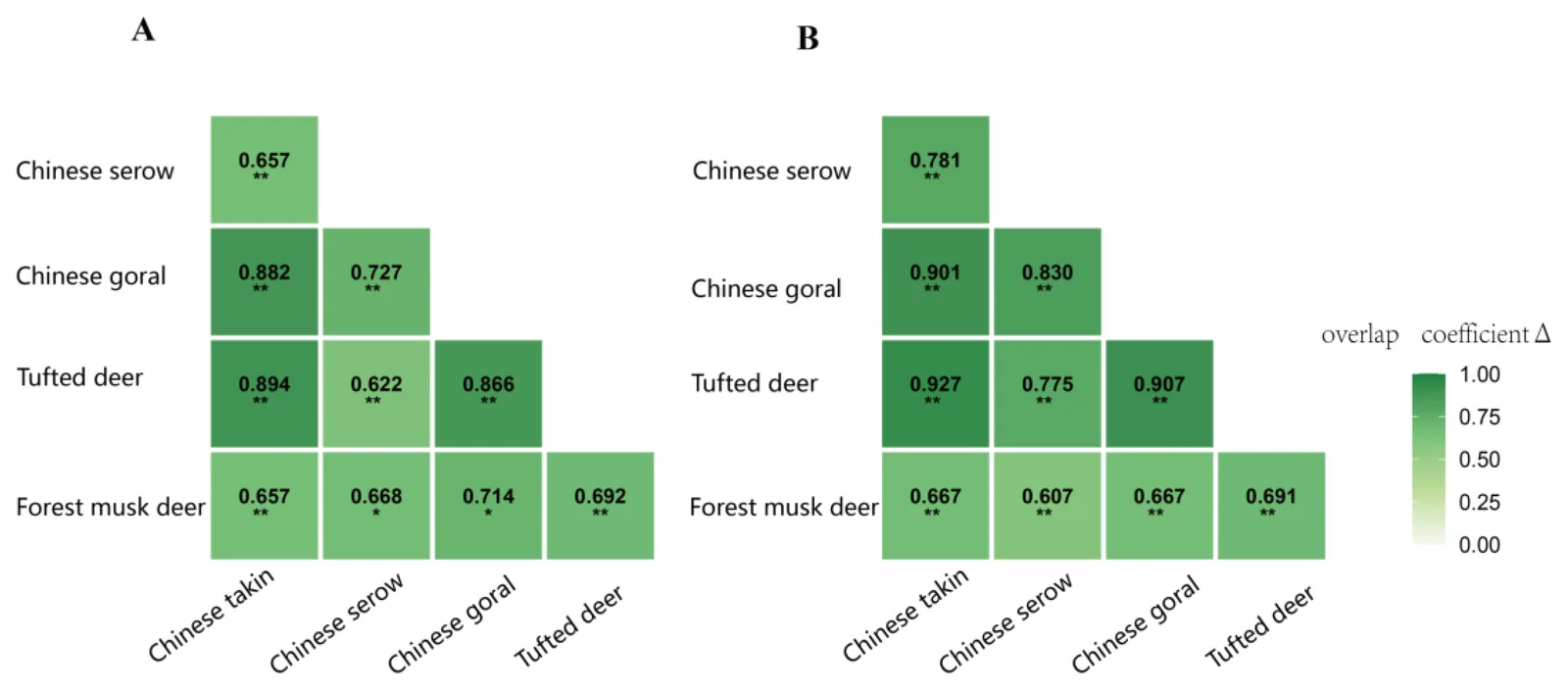
Temporal overlap of the five ungulate species in the Tangjiahe National Nature Reserve during the cold (**A**) and warm (**B**) seasons. * indicates *p* < 0.05, and ** indicates *p* < 0.01.

**Figure 6:**
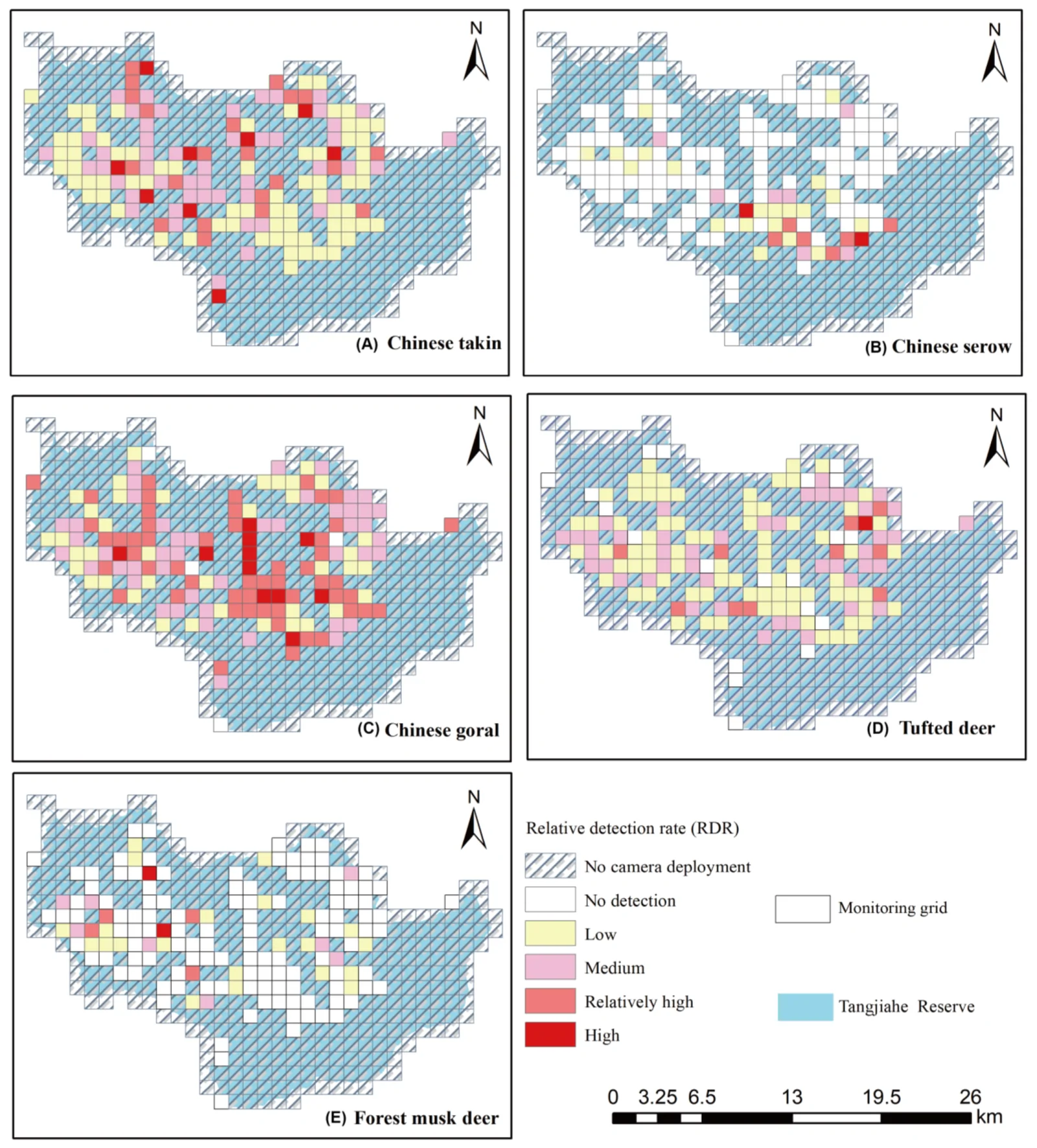
Relative detection rate (RDR) of five ungulate species across 1-km grid cells in the study area. RDR was standardized by grid-level effective camera-days to account for uneven sampling effort. (**A**) Chinese takin; (**B**) Chinese serow; (**C**) Chinese goral; (**D**) tufted deer; (**E**) forest musk deer. Grids without camera deployment are indicated by hatching; grids with cameras but without species detections are defined as “No detection”.

**Figure 7:**
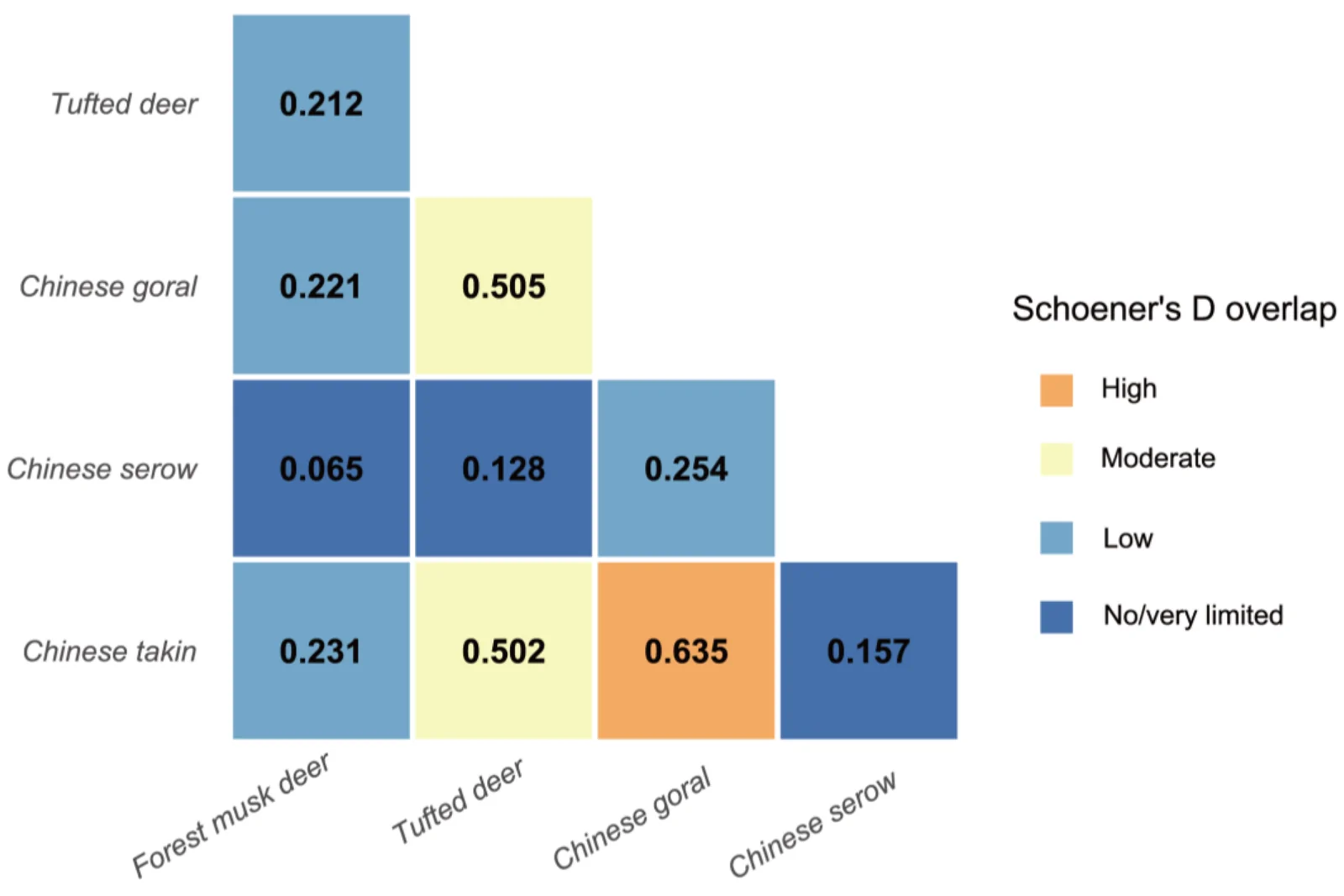
Spatial overlap of the five ungulate species in the Tangjiahe National Nature Reserve.

**Table 1:** Relative detection index (RAI) of the five ungulate species in the Tangjiahe National Nature Reserve, 2022–2024.

Species	2022 RAI	2023 RAI	2024 RAI	Overall RAI
MAMMALIA				
CETARTIODACTYLA				
Bovidae				
Chinese takin (*Budorcas tibetana*)	11.915	8.022	18.811	15.110
Chinese serow (*Capricornis milneedwardsii*)	0.080	0.100	0.128	0.111
Chinese goral (*Naemorhedus griseus*)	6.301	4.707	11.356	8.886
Cervidae				
Tufted deer (*Elaphodus cephalophus*)	3.791	2.028	4.881	4.074
Moschidae				
Forest musk deer (*Moschus berezovskii*)	0.033	0.050	0.071	0.058

Note: RAI is defined as independent detection events per 100 effective camera-days. Annual RAI uses year-specific effective camera-days as the denominator; overall RAI uses cumulative total effective camera-days across 2022–2024 as the denominator, and is a weighted average rather than the arithmetic mean of annual values.

**Table 2:** Diel activity-related indices of the five ungulate species in the Tangjiahe Reserve.

Species	Shannon–Wiener Diversity Index (H)	Maximum Shannon–Wiener Diversity Index (Hmax)	Evenness Index (J)	Daily Activity Difference Index (DDI)	Diurnality Index (DI) (95% Bootstrap CI)
Chinese takin	3.057	3.178	0.962	0.038	0.320 (0.308–0.332)
Chinese serow	3.085	3.178	0.971	0.029	−0.106 (−0.236–0.027)
Chinese goral	3.139	3.178	0.988	0.012	0.190 (0.174–0.206)
Tufted deer	3.080	3.178	0.969	0.031	0.283 (0.260–0.306)
Forest musk deer	2.766	3.178	0.870	0.130	−0.253 (−0.423–−0.084)

Note: Hmax is a theoretical maximum Shannon–Wiener diversity constant calculated as ln(S), where S = 24, corresponding to 1-h time bins across the full 24-h diel cycle; this value is identical for all species and is not an empirical observation.

**Table 3:** Summary of species-specific generalized additive model (GAM) results for environmental predictors and interannual variation.

Species	Deviance Explained (%)	Elevation	Slope	Aspect	LUCC	Distance to Water	Distance to Roads	Year
Chinese takin	18.5	0.016	0.133	0.005	<0.001	0.209	<0.001	<0.001
Chinese serow	53.4	<0.001	0.021	0.286	<0.001	0.013	<0.001	0.176
Chinese goral	20.2	<0.001	0.268	<0.001	0.003	0.096	<0.001	<0.001
Tufted deer	18.6	<0.001	0.138	<0.001	0.097	0.245	<0.001	<0.001
Forest musk deer	32.1	0.040	0.159	0.010	0.249	0.011	0.002	0.004

Notes: Values are term-level *p* values obtained from likelihood-ratio tests comparing the six-predictor full model, including elevation, with a reduced model excluding the focal term. LUCC, land use/land cover. Year represents the overall interannual effect across the 2022–2024 monitoring period. Deviance explained refers to the full species-specific model.

## Data Availability

The data presented in this study are available on request from the corresponding author due to the sensitive nature of wildlife monitoring locations and species occurrence information within the protected area.

## Supplementary Materials

The following supporting information can be downloaded at: https://www.mdpi.com/article/10.3390/biology15191729/s1, Figure S1: GAM responses of five sympatric ungulate species to elevation; Figure S2: GAM responses of five sympatric ungulate species to slope; Figure S3: GAM responses of five sympatric ungulate species to aspect; Figure S4: GAM responses of five sympatric ungulate species to land-use/land-cover (LUCC) category; Figure S5: AM responses of five sympatric ungulate species to distance to water; Figure S6: GAM responses of five sympatric ungulate species to distance to road; Table S1: Summary of camera-trapping sampling effort across 2022–2024 in Tangjiahe National Nature Reserve; Table S2: nnual and total independent detections, effective camera-days, and relative abundance index (RAI) for five ungulate species during 2022–2024; Table S3: Summary of camera-site-year observations and zero detections used in the species-specific GAM analyses.

## Abbreviations

The following abbreviations are used in this manuscript:

RAI	The relative abundance index
DI	The diurnality index
DDI	The daily activity difference index
RDR	The relative detection rate
