# Supplementary material for: Spatiotemporal Patterns of Five Sympatric Ungulates in the Tangjiahe National Nature Reserve, Sichuan, China
Source: Biology (Basel). 2026 Oct 1;15(19):1729. doi: 10.3390/biology15191729 (PMC13663518; doi:10.3390/biology15191729)
Supplement: Supplementary file 1 [file biology-15-01729-s001.zip › biology-4496195-supplementary.pdf]

Table S1. Summary of camera-trapping sampling effort across 2022-2024 in Tangjiahe National Nature Reserve.

Year	Unique camera stations	Camera-deployment events	Effective camera-days	Mean duration per deployment (days)
2022	221	163	33790	207.3
2023	69	158	27960	177.0
2024	302	471	82708	175.6
Total / Mean	592	792	144458	182.4

Note: Unique camera stations refers to spatially distinct physical locations, whereas camera-deployment events counts all installations including re-deployments at the same station after maintenance or relocation. Effective camera-days are summed over all deployment events after excluding invalid periods.

Table S2. Annual and total independent detections, effective camera-days, and relative abundance index (RAI) for five ungulate species during 2022–2024.

species	year	Independent detections	Trap-days	RAI
Chinese takin	2022	4026	33790	11.91476768
Chinese goral	2022	2129	33790	6.300680675
Chinese serow	2022	27	33790	0.079905297
Forest musk deer	2022	11	33790	0.03255401
Tufted deer	2022	1281	33790	3.791062445
Chinese takin	2023	2243	27960	8.022174535
Chinese goral	2023	1316	27960	4.706723891
Chinese serow	2023	28	27960	0.100143062
Forest musk deer	2023	14	27960	0.050071531
Tufted deer	2023	567	27960	2.027896996
Chinese takin	2024	15558	82708	18.81075591
Chinese goral	2024	9392	82708	11.35561252
Chinese serow	2024	106	82708	0.128161726
Forest musk deer	2024	59	82708	0.0713353
Tufted deer	2024	4037	82708	4.881027228
Chinese takin	Total	21827	144458	15.10958202
Chinese goral	Total	12837	144458	8.886319899
Chinese serow	Total	161	144458	0.111451079
Forest musk deer	Total	84	144458	0.058148389
Tufted deer	Total	5885	144458	4.073848454

Table S3. Summary of camera-site-year observations and zero detections used in the species-specific GAM analyses.

Species	Total camera-site-years	Site-years with detection	Zero-detection site-years	Zero-detection proportion (%)	Total independent detection events
Chinese takin	792	760	32	4.04	21827
Chinese serow	792	72	720	90.91	161
Chinese goral	792	703	89	11.24	12837
Tufted deer	792	535	257	32.45	5885
Forest musk deer	792	53	739	93.31	84

Note: "Site-years with detection" indicates camera-site-year observations with at least one independent detection event for the focal species, whereas "zero-detection site-years" indicates observations retained in the model with a response value of zero. Zero-detection proportion was calculated as the number of zero-detection site-years divided by the total number of camera-site-year observations.

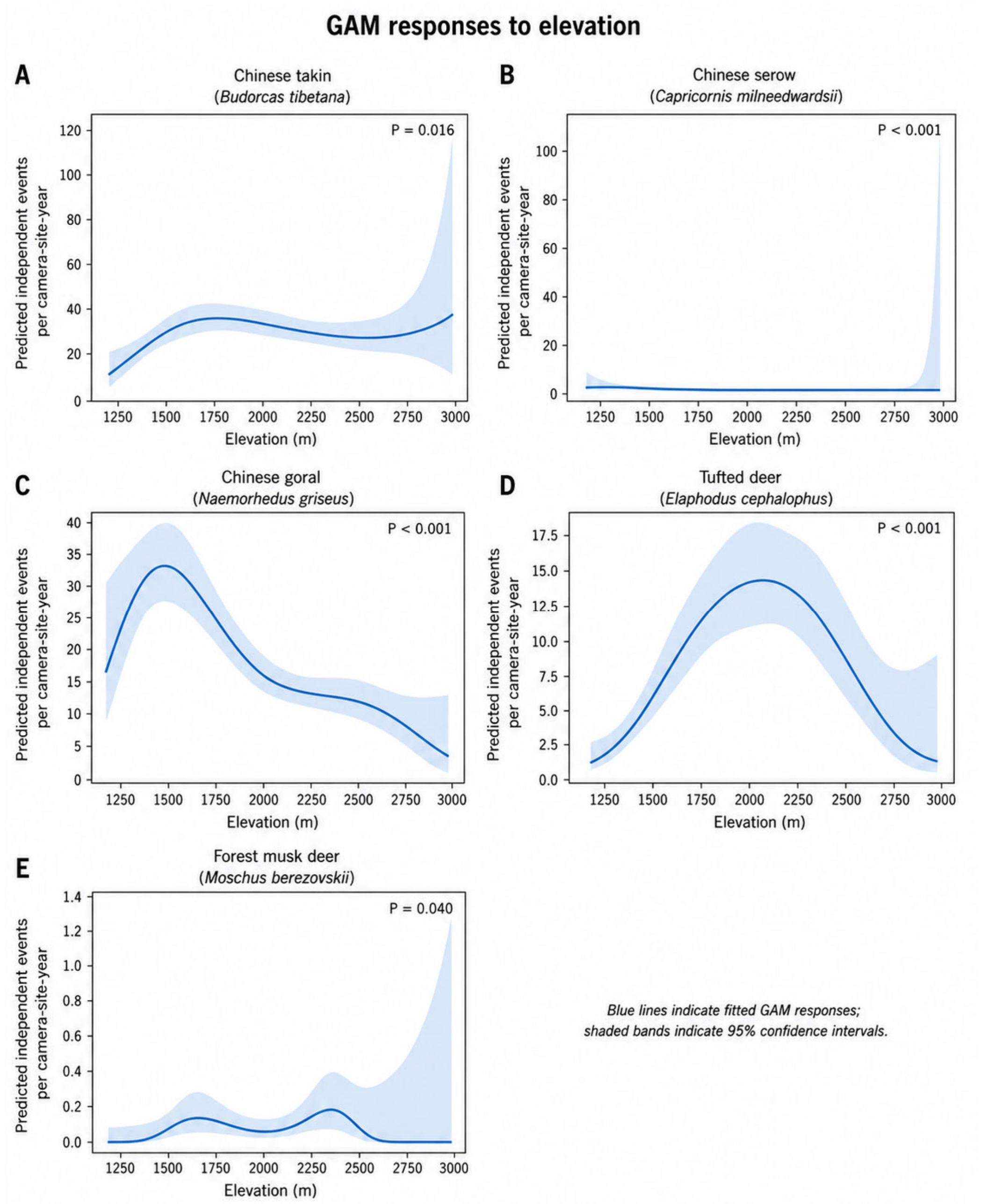

Figure S1. GAM responses of five sympatric ungulate species to elevation. Generalized additive model (GAM) responses of Chinese takin (*Budorcas tibetana*), Chinese serow (*Capricornis milneedwardsii*), Chinese goral (*Naemorhedus griseus*), tufted deer (*Elaphodus cephalophus*), and forest musk deer (*Moschus berezovskii*) to elevation. Blue lines indicate fitted GAM responses, and shaded bands indicate 95% confidence intervals. P-values are shown in each panel.

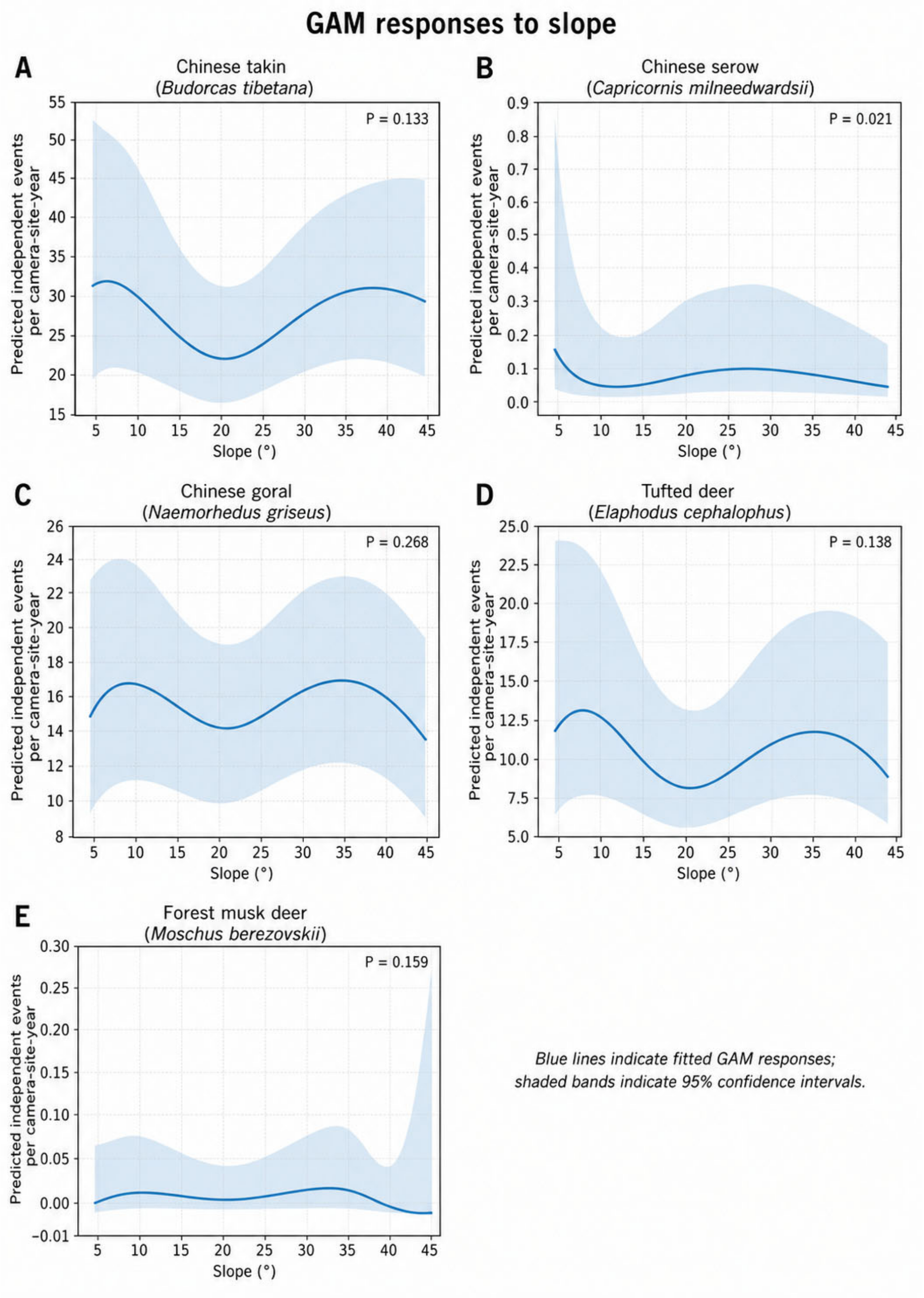

Figure S2. GAM responses of five sympatric ungulate species to slope. Generalized additive model (GAM) responses of Chinese takin (*Budorcas tibetana*), Chinese serow (*Capricornis milneedwardsii*), Chinese goral (*Naemorhedus griseus*), tufted deer (*Elaphodus cephalophus*), and forest musk deer (*Moschus berezovskii*) to slope. Blue lines indicate fitted GAM responses, and shaded bands indicate 95% confidence intervals. P-values are shown in each panel.

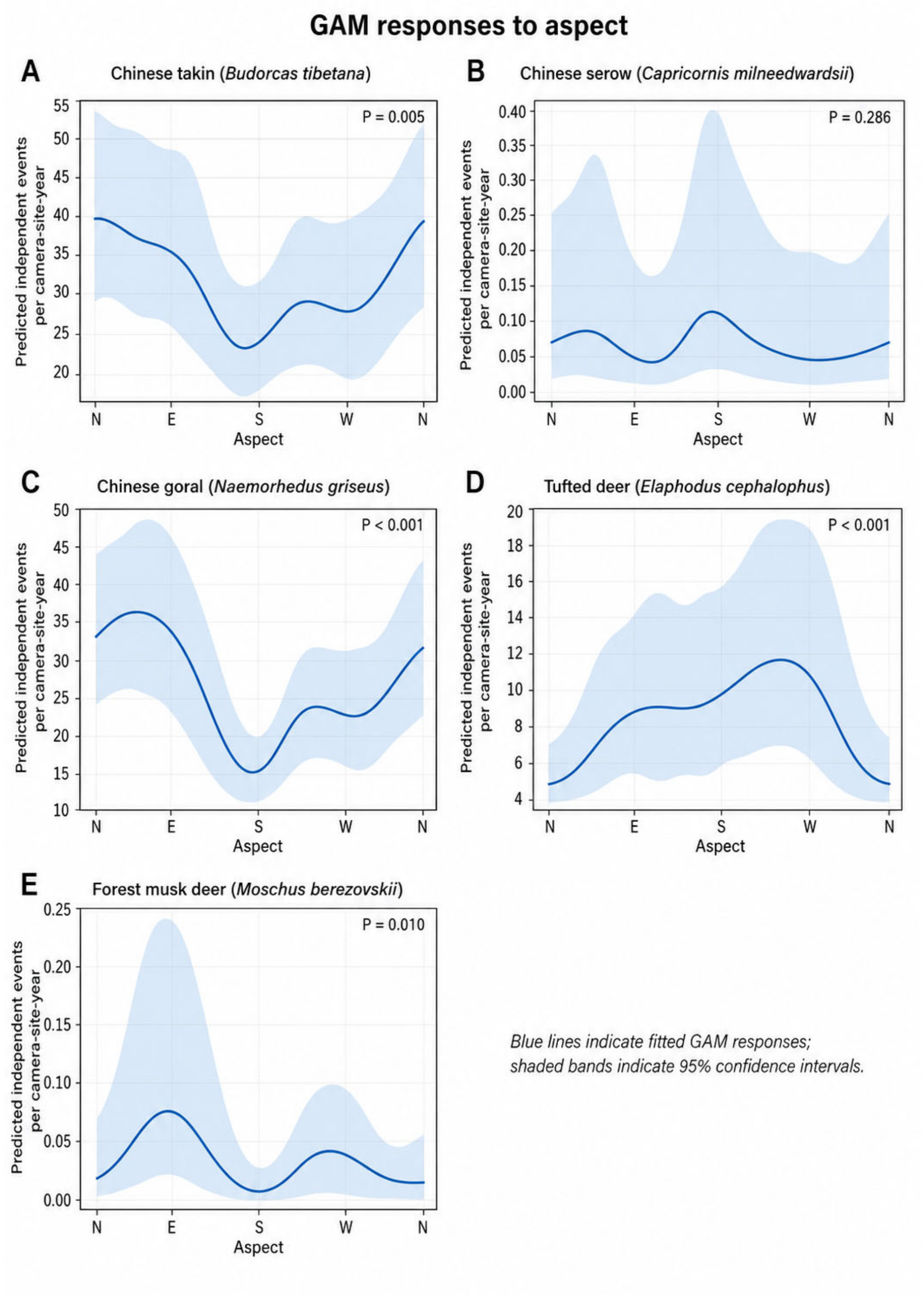

Figure S3. GAM responses of five sympatric ungulate species to aspect. Generalized additive model (GAM) responses of Chinese takin (*Budorcas tibetana*), Chinese serow (*Capricornis milneedwardsii*), Chinese goral (*Naemorhedus griseus*), tufted deer (*Elaphodus cephalophus*), and forest musk deer (*Moschus berezovskii*) to aspect. Blue lines indicate fitted GAM responses, and shaded bands indicate 95% confidence intervals. P-values are shown in each panel.

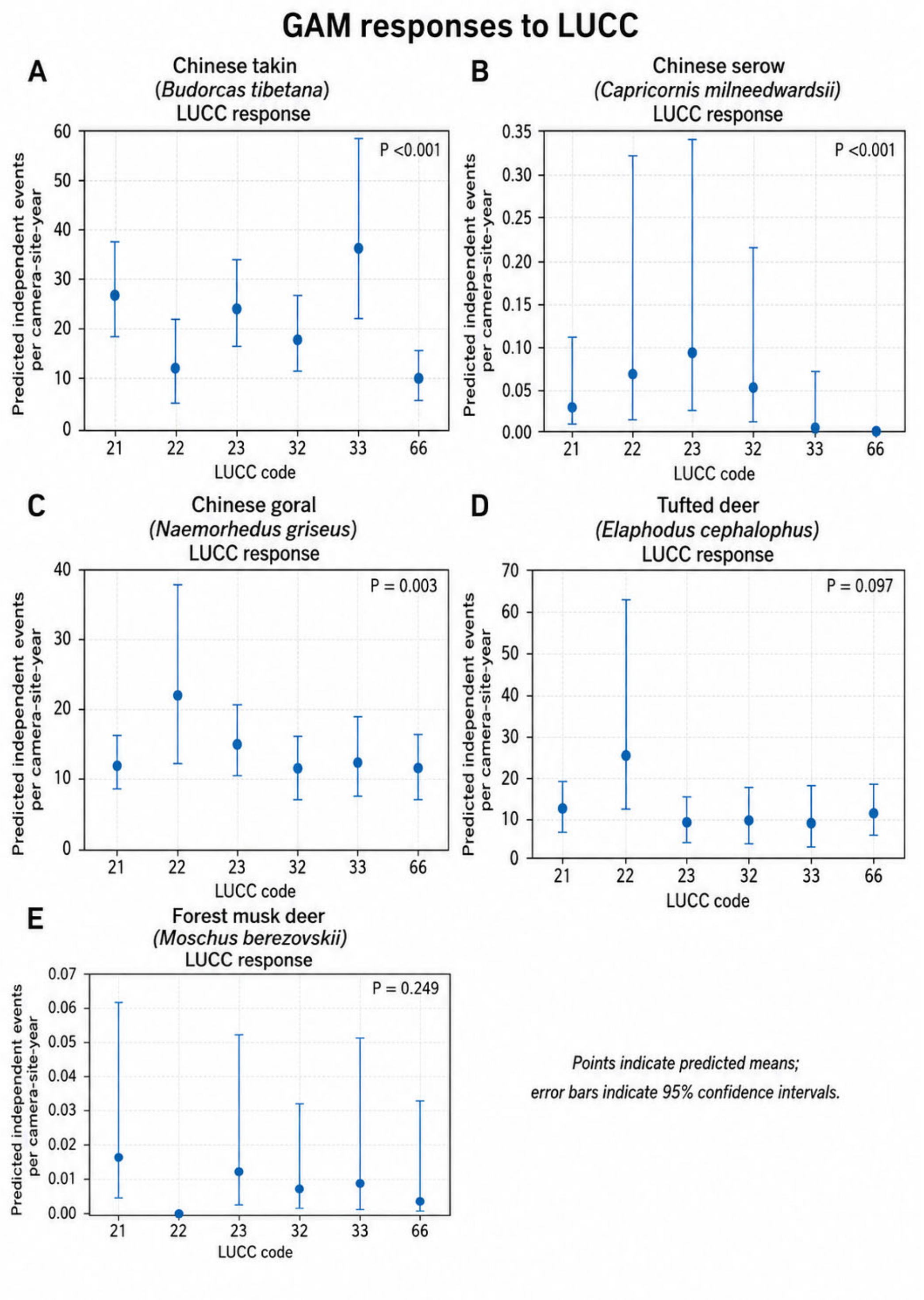

Figure S4. GAM responses of five sympatric ungulate species to land-use/land-cover (LUCC) category. Generalized additive model (GAM) responses of Chinese takin (*Budorcas tibetana*), Chinese serow (*Capricornis milneedwardsii*), Chinese goral (*Naemorhedus griseus*), tufted deer (*Elaphodus cephalophus*), and forest musk deer (*Moschus berezovskii*) to land-use/land-cover (LUCC) category. Points indicate predicted means, and error bars indicate 95% confidence intervals. P-values are shown in each panel.

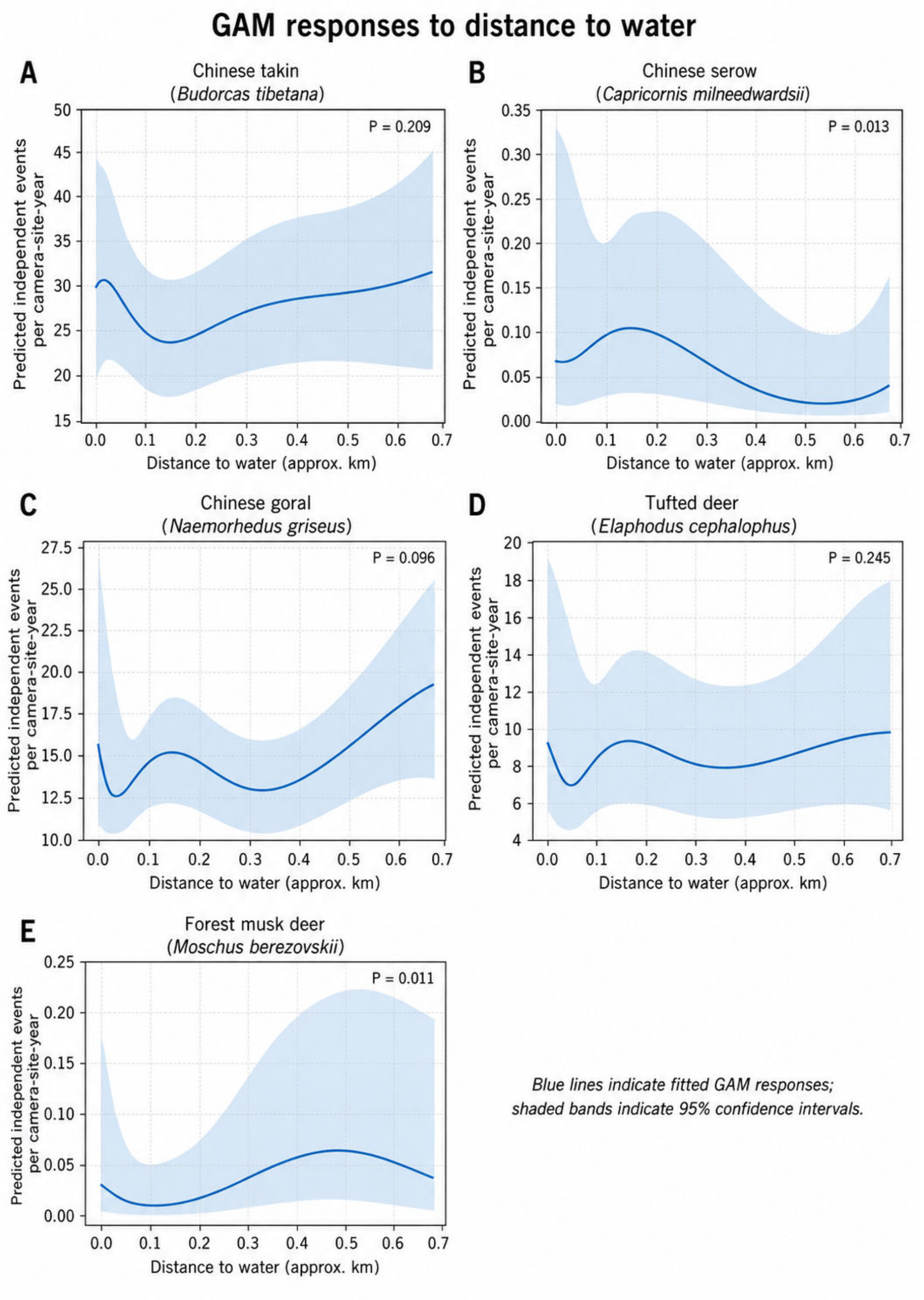

Figure S5. GAM responses of five sympatric ungulate species to distance to water. Generalized additive model (GAM) responses of Chinese takin (*Budorcas tibetana*), Chinese serow (*Capricornis milneedwardsii*), Chinese goral (*Naemorhedus griseus*), tufted deer (*Elaphodus cephalophus*), and forest musk deer (*Moschus berezovskii*) to distance to water. Blue lines indicate fitted GAM responses, and shaded bands indicate 95% confidence intervals. P-values are shown in each panel.

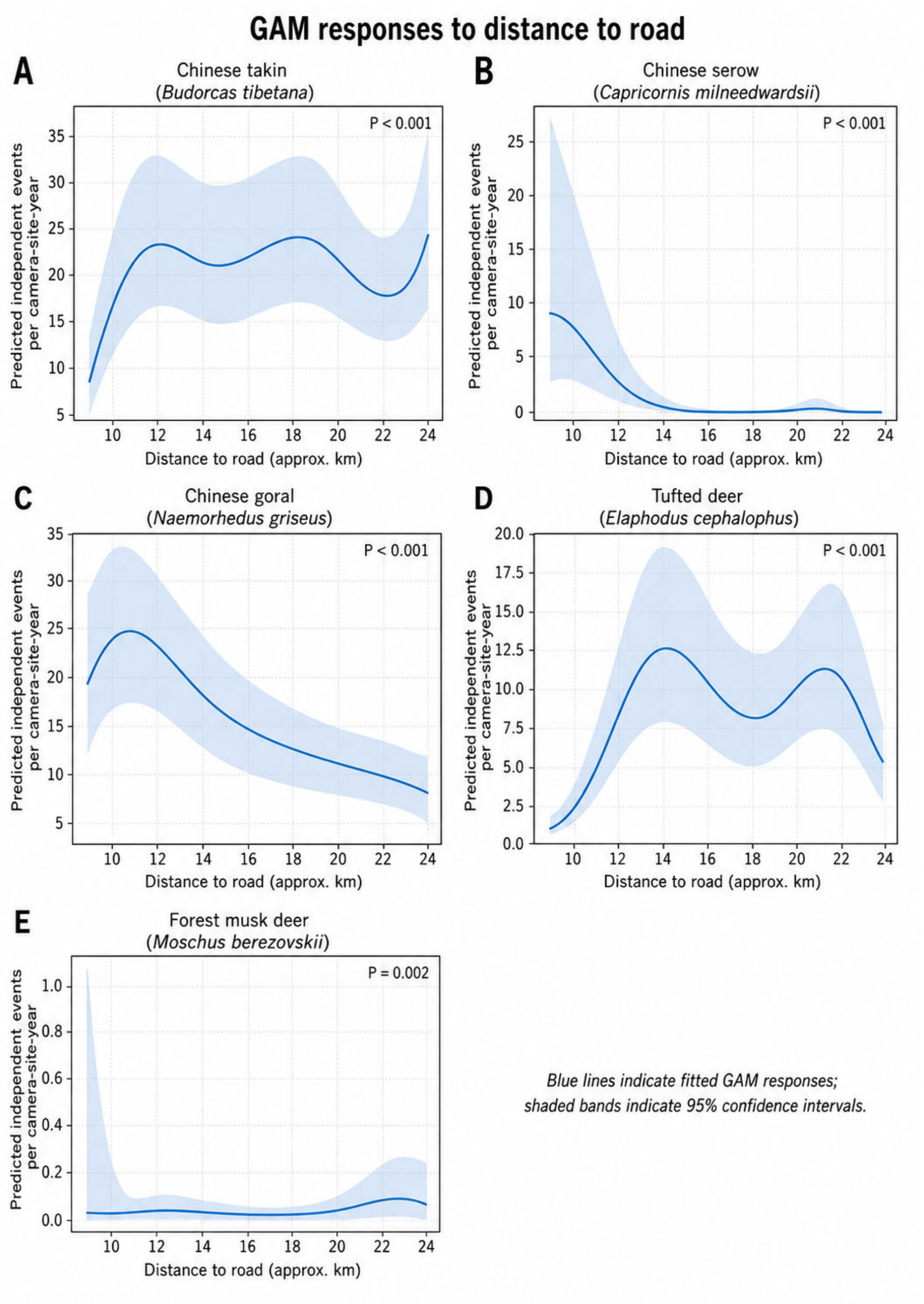

Figure S6. GAM responses of five sympatric ungulate species to distance to road. Generalized additive model (GAM) responses of Chinese takin (*Budorcas tibetana*), Chinese serow (*Capricornis milneedwardsii*), Chinese goral (*Naemorhedus griseus*), tufted deer (*Elaphodus cephalophus*), and forest musk deer (*Moschus berezovskii*) to distance to road. Blue lines indicate fitted GAM responses, and shaded bands indicate 95% confidence intervals. P-values are shown in each panel.
